# A Single-Molecule Strategy to Capture Non-native Intramolecular and Intermolecular Protein Disulfide Bridges

**DOI:** 10.1021/acs.nanolett.2c00043

**Published:** 2022-05-12

**Authors:** Marc Mora, Stephanie Board, Olivier Languin-Cattoën, Laura Masino, Guillaume Stirnemann, Sergi Garcia-Manyes

**Affiliations:** † Department of Physics, Randall Centre for Cell and Molecular Biophysics and London Centre for Nanotechnology, King’s College London, Strand, WC2R 2LS London, United Kingdom; ‡ Single Molecule Mechanobiology Laboratory, The Francis Crick Institute, 1 Midland Road, London NW1 1AT, London United Kingdom; § CNRS Laboratoire de Biochimie Théorique, Institut de Biologie Physico-Chimique, Université Paris Diderot, Sorbonne Paris Cité, PSL Research University, 13 rue Pierre et Marie Curie, 75005 Paris, France; ∥ Structural Biology Science Technology Platform, The Francis Crick Institute, 1 Midland Road London, NW1 1AT, United Kingdom

**Keywords:** protein nanomechanics, protein mechanochemistry, non-native disulfide bonds, single-molecule force spectroscopy, atomic force microscopy (AFM), protein folding

## Abstract

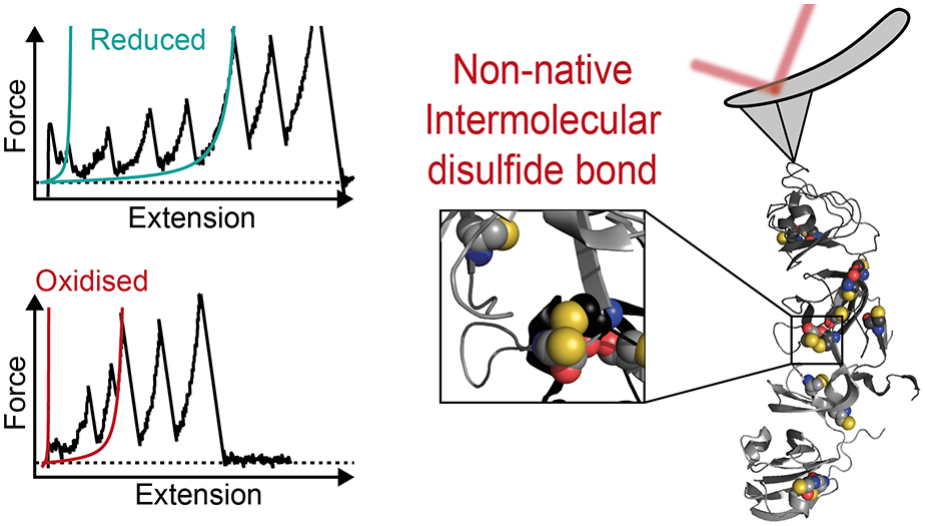

Non-native disulfide bonds are dynamic covalent bridges that form post-translationally between two cysteines within the same protein (intramolecular) or with a neighboring protein (intermolecular), frequently due to changes in the cellular redox potential. The reversible formation of non-native disulfides is intimately linked to alterations in protein function; while they can provide a mechanism to protect against cysteine overoxidation, they are also involved in the early stages of protein multimerization, a hallmark of several protein aggregation diseases. Yet their identification using current protein chemistry technology remains challenging, mainly because of their fleeting reactivity. Here, we use single-molecule spectroscopy AFM and molecular dynamics simulations to capture both intra- and intermolecular disulfide bonds in γD-crystallin, a cysteine-rich, structural human lens protein involved in age-related eye cataracts. Our approach showcases the power of mechanical force as a conformational probe in dynamically evolving proteins and presents a platform to detect non-native disulfide bridges with single-molecule resolution.

## Introduction

Native disulfide bridges—strong covalent bonds formed between two close cysteine residues—have been typically shown to provide structural stabilization to the folded conformation of proteins.^1,2^ In addition to those disulfides with a clear structural—static—function, other intramolecular disulfide bonds display a dynamic behavior in virtue of their reversible thiol/disulfide chemistry, a mechanism that is collectively related to a myriad of redox-mediated signaling cellular processes,^3−5^ and tightly modulated by the fluctuating redox properties of the environment and by dedicated oxidoreductase enzymes that ensure overall redox homeostasis.^6^ A further layer of complexity is added when solvent-exposed cysteines establish intermolecular disulfide bonds with a neighboring protein.^7−9^ While intermolecular disulfide bonds play decisive functional roles—for example, they work as protective mechanisms against cysteine irreversible overoxidation^10,11^ but are also involved in a large number of aggregation misfolding reactions^12−14^—their extremely fleeting nature makes them challenging to detect experimentally. Several techniques, such as NMR have attempted to capture their presence in solution,^15^ and a handful of crystal structures have provided the snapshots of a subset of (long-lived) stable conformations.^16^ However, their markedly fast reactivity, underpinning their ability to multimerize in a (often) rather uncontrolled way, calls for the development of new experimental approaches enabling direct detection of non-native disulfide bonds.

Single-molecule force spectroscopy AFM has emerged as a powerful technique to identify intramolecular disulfide bonds,^17^ which work as strong intramolecular staples. Typically, stretching a protein with an AFM extends it to almost its full contour length.^18^ The presence of an intramolecular disulfide bond effectively shortcuts the protein, limiting its extensibility.^19^ Hence, a shorter than expected increment of the protein’s contour length can be considered as a reliable reporter of the presence of intramolecular disulfide bridges, as demonstrated in titin,^20,21^ calmodulin binding domain,^22^ FimG domain^23^ and cell adhesion molecule domains.^24^ Recent nanomechanical experiments also captured the dynamics of reduction and reformation of individual intramolecular disulfides by measuring the changes in protein extensibility over time.^25−30^ Despite the unquestionable progress, this single-molecule approach is still not suitable to capture intermolecular, non-native disulfide bonds, notably because of the natural requirement of establishing a physical connection between (at least) two contacting proteins in the assay. Here, using a combination of protein engineering techniques (based on the rational design of polyproteins whereby the number of adjacent monomers prone to oxidation is precisely controlled), molecular dynamics (MD) simulations, and single-molecule force spectroscopy experiments, we develop an integrated single-molecule experimental approach able to capture both non-native inter- and intramolecular individual disulfide bonds, and characterize their dynamics under biologically mimicking redox conditions. We showcase our proof-of-principle experimental platform to study the complex redox reactivity of human γD-crystallin (γDc, a key human structural protein in the eye lens containing 6 reduced native cysteines), the aggregation of which^31−33^—intricately related to cysteine oxidation^32^—gives rise to the high-molecular weight protein aggregates that hallmark the eye’s cataract disease.^34,35^ Noteworthy, the ability to identify and trap non-native intermolecular disulfide bonds fingerprints a conformational change, involving the spontaneous and relatively frequent excursion of the native γD-crystallin to a well-defined intermediate conformation, that exposes to the solvent those natively cryptic (and thus unreactive) cysteines, becoming suddenly reactive. Besides enabling direct identification of non-native (intramolecular and intermolecular) disulfide bonds, our single-molecule strategy unambiguously captures disulfide-bond mediated protein dimerization and provides a direct probe of the subtle interplay between the redox status of a protein and its conformational dynamics.

## Results

**Figure 1:**
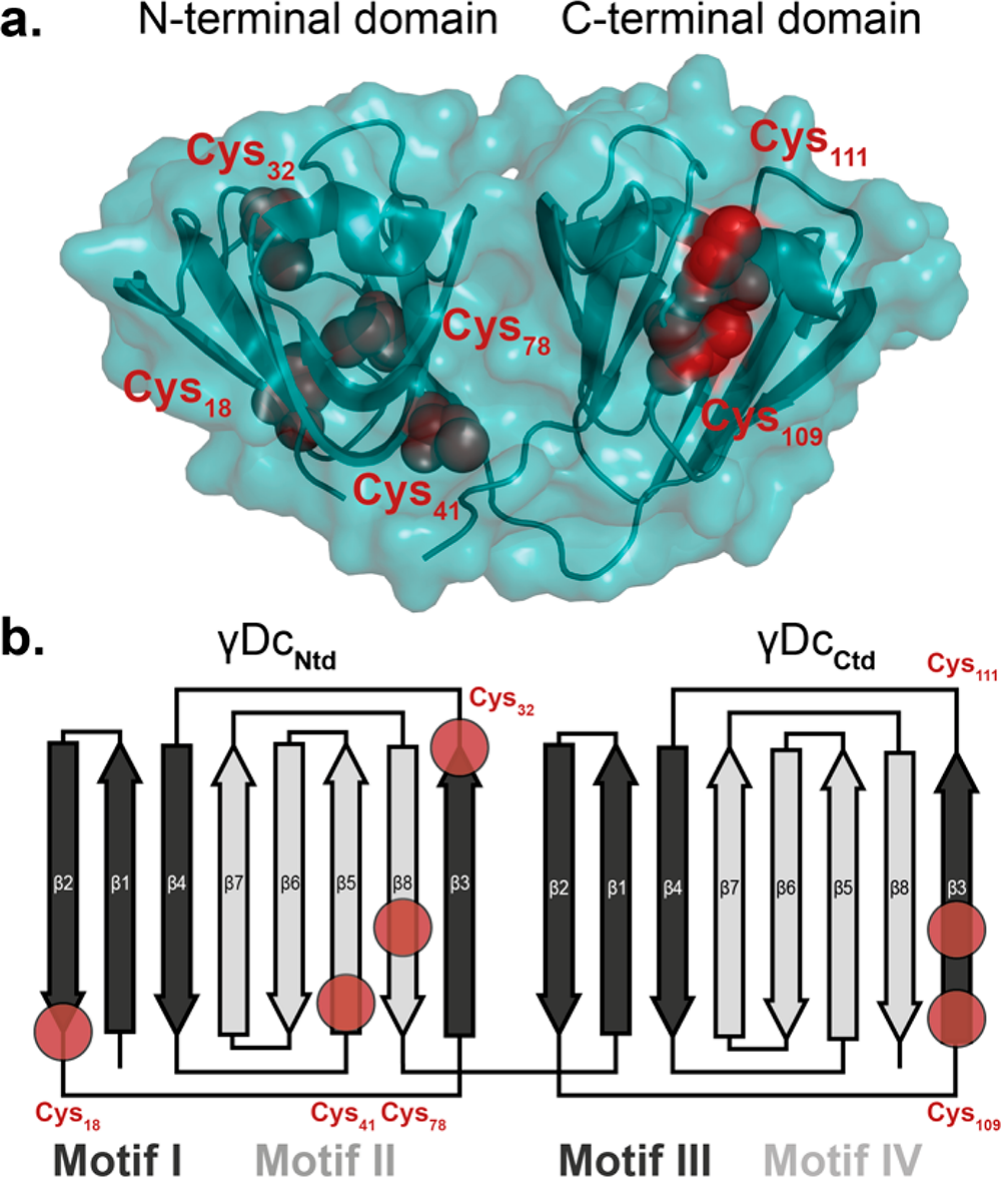
Cysteine content in γD-crystallin. (a) Cartoon representation of the γDc structure (PDB: 1HK0) harboring six reduced cysteines (red) unevenly distributed between the two protein termini. The two globular domains (N-terminal domain, Ntd; C-terminal domain, Ctd) fold independently and are joined by an unstructured 7-amino acid linker. (b) Schematic representation of the 4 characteristic Greek key motifs of the γDc (motifs I–IV), consisting of two intercalated antiparallel β-sheet motifs in each termini. Each red circle marks the location of the cysteines within the protein structure.

**Figure 2:**
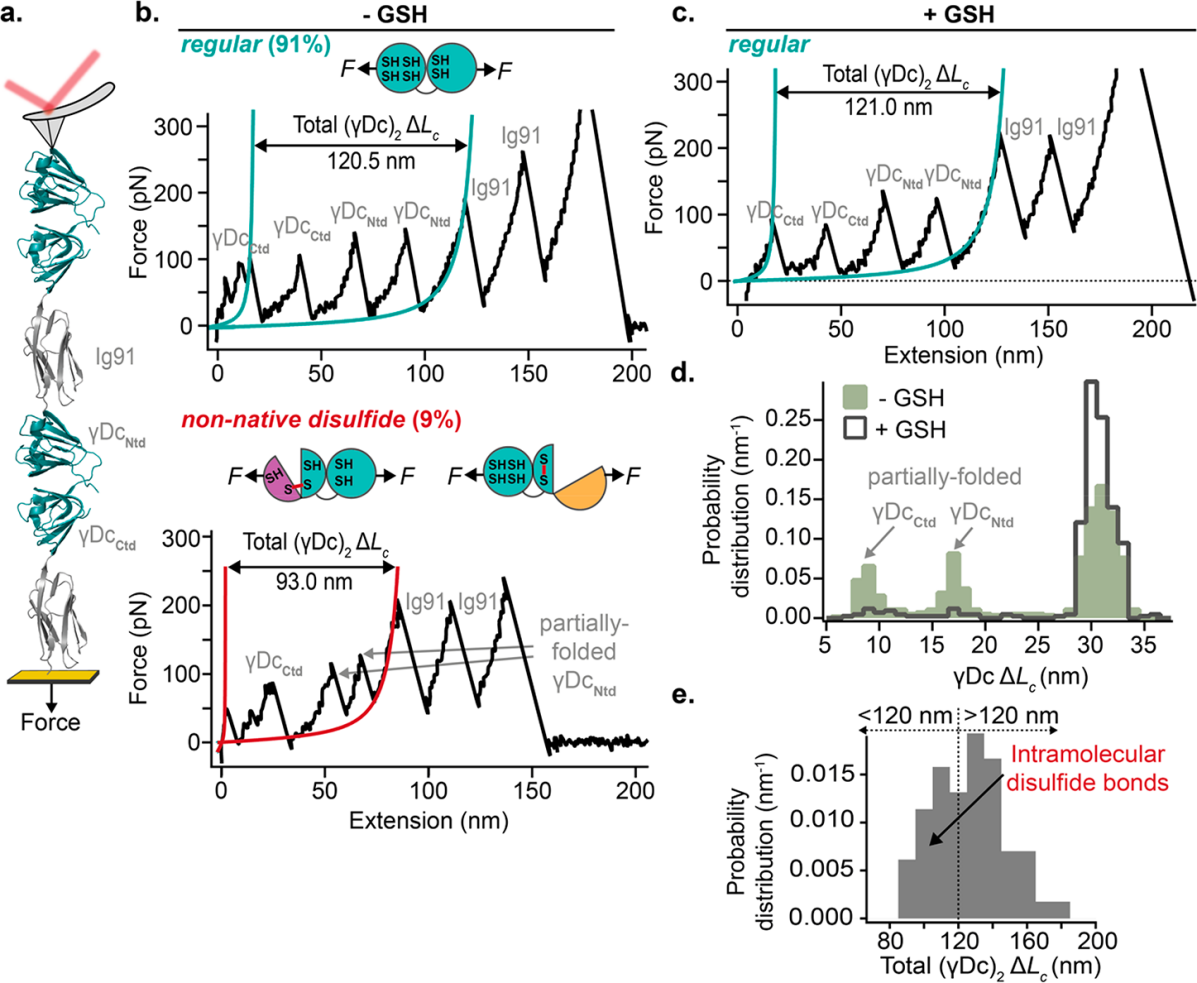
Capturing intramolecular non-native disulfide bonds. (a) Schematics of the single-molecule AFM experiment, whereby a single engineered (Ig91−γDc)_2_ polyprotein is stretched between an AFM cantilever and a gold-coated cover slide. (b) Representative force–extension unfolding trajectories of the (Ig91−γDc)_2_ polyprotein in the absence of reducing agent. The most common *regular* trajectory (top) displays the uncomplicated unfolding of the two mechanically weaker Ctds first, followed by the unfolding of the two Ntds, releasing a total Δ*L*_c_ of ∼120 nm (turquoise WLC fits). The last two unfolding events correspond to the unfolding of the Ig91 marker domains. The shorter force–extension trace (bottom) shows an overall reduction in the (γDc)_2_ extension (<120 nm), reminiscent of intramolecular non-native disulfide bridge(s) formation. (c) In the presence of 1 mM of deprotonated GSH, only the *regular* phenotype is observed. (d) Histogram comparing the frequency of observed increment in contour-length in γDc corresponding to the stretching of (γDc–Ig91)_2_ polyprotein in the presence and absence of GSH [*n* = 564 unfolding events without GSH (green) and *n* = 422 unfolding events with GSH (empty gray)]. (e) Histogram of the total increase in contour length, (γDc)_2_ Δ*L*_c_, when pulling (Ig91−γDc)_2_ in the absence of a reducing agent (*n* = 114 unfolding trajectories).

Human γD-crystallin (γDc) is the third most abundant crystallin in the human lens,^36^ which folds in the characteristic crystallin two-domain structure (Figure 1a). Despite its high degree of structural symmetry provided by its characteristic two Greek-key motifs, its cysteine content is not evenly distributed among the terminals. While the N-terminal domain (Ntd) harbors 4 cysteines, the C-terminal domain (Ctd) contains only two cysteines, arranged in a CXC motif, Figure 1b. In neither termini does the crystallized native structure show the presence of a disulfide bridge (PDB: 1HK0). Consequently, mechanical unfolding and stretching of a single γDc monomer with an AFM should uncomplicatedly elicit the whole length of the protein. To test this hypothesis, we first engineered a polyprotein construct whereby two γDc monomers are intercalated with the Ig91 marker protein, (Ig91−γDc)_2_ (Figure 2a). This strategy enables the marker protein not only to serve as an internal mechanical fingerprint (the mechanical properties of the Ig91 protein have been well-characterized,^18^Supporting Figures 1 and 2) but also to avoid any physical interaction between the two γDc monomers. Stretching an individual (Ig91−γDc)_2_ polyprotein at a constant velocity of 400 nm s^–1^ with an AFM commonly resulted in unfolding trajectories with (up to) four unfolding peaks prior to the two unfolding peaks of the Ig91 markers (Figure 2b, top). Each of the (Ntd and Ctd) termini within each γDc monomer unfolded independently, and the mechanical unfolding of the full (Ig91−γDc)_2_ construct quantitatively agreed in terms of unfolding forces (*F*_Ntd_ = 131 ± 15 pN; *F*_Ctd_ = 100 ± 19 pN) and contour length increments (Δ*L*_c__,Ntd_ = 29.0 ± 1.1 nm; Δ*L*_c__,Ctd_ = 29.7 ± 1.0 nm) with the independent mechanical characterization of each individual terminus (Supporting Figure 3). Consequently, in the full (Ig91−γDc)_2_ typical unfolding trajectory, the first two peaks (Figure 2b, top) corresponded to the unfolding of the mechanically weaker Ctds, followed by the unfolding of the two Ntds, displaying higher mechanical stability, before the unfolding of the higher mechanical stability Ig91 markers.

As expected, in the vast majority of the unfolding traces (91%) (Figure 2b, top) the total full γDc length Δ*L*_c_ ∼120 nm is released upon unfolding, since each γDc terminal contributes with ∼30 nm (4 termini × 30 nm = 120 nm, Supporting Figure 4). However, a few yet significant (9%) unfolding trajectories showed a marked reduction in the total (γDc)_2_ Δ*L*_c_ unfolding length, (γDc)_2_ Δ*L*_c_ < 120 nm (Figure 2b, bottom and Supporting Figures 5 and 6). To unambiguously resolve if such molecular shortening is the result of the formation of non-native disulfide bridges, we reproduced the experiments in a reduced environment (1 mM of deprotonated glutathione). Pulling the same (Ig91−γDc)_2_ polyprotein (Figure 2a) in the presence of GSH confirmed the absence of trajectories featuring a reduction in the total (γDc)_2_ extension (Figure 2c), strongly suggesting that the shorter unfolding trajectories obtained under oxidizing (PBS) conditions (Figure 2b, bottom) very likely correspond to the formation of (non-native) intramolecular disulfide bonds that are not found in the native crystal structure (Figure 1). Close inspection to the contour length histogram associated with crystallin unfolding (Figure 2d) revealed a predominant peak at ∼30 nm, likely corresponding to the all-or-none unfolding of each individual domain, in addition to two extra lower occurrence peaks at shorter lengths (Δ*L*_c_ ∼15 and ∼9 nm) that correspond to the unfolding of well-defined partially folded conformations occurring in the Ntd (Δ*L*_c_ ∼15 nm and ∼130 pN) or the Ctd (Δ*L*_c_ ∼9 nm and ∼155 pN), clearly distinguishable when pulling both γDc terminals independently (Supporting Figure 3). Remarkably, the relative abundance of both peaks is dramatically reduced under reducing conditions, while the probability of observing a ∼30 nm full-length unfolding event is instead increased. Combined, these experiments suggest that those shorter trajectories (Figure 2b, bottom) leading to protein extensions shorter than ∼120 nm (Figure 2e) are directly related to the mechanical intermediate conformations that largely resolve upon GSH addition.

**Figure 3:**
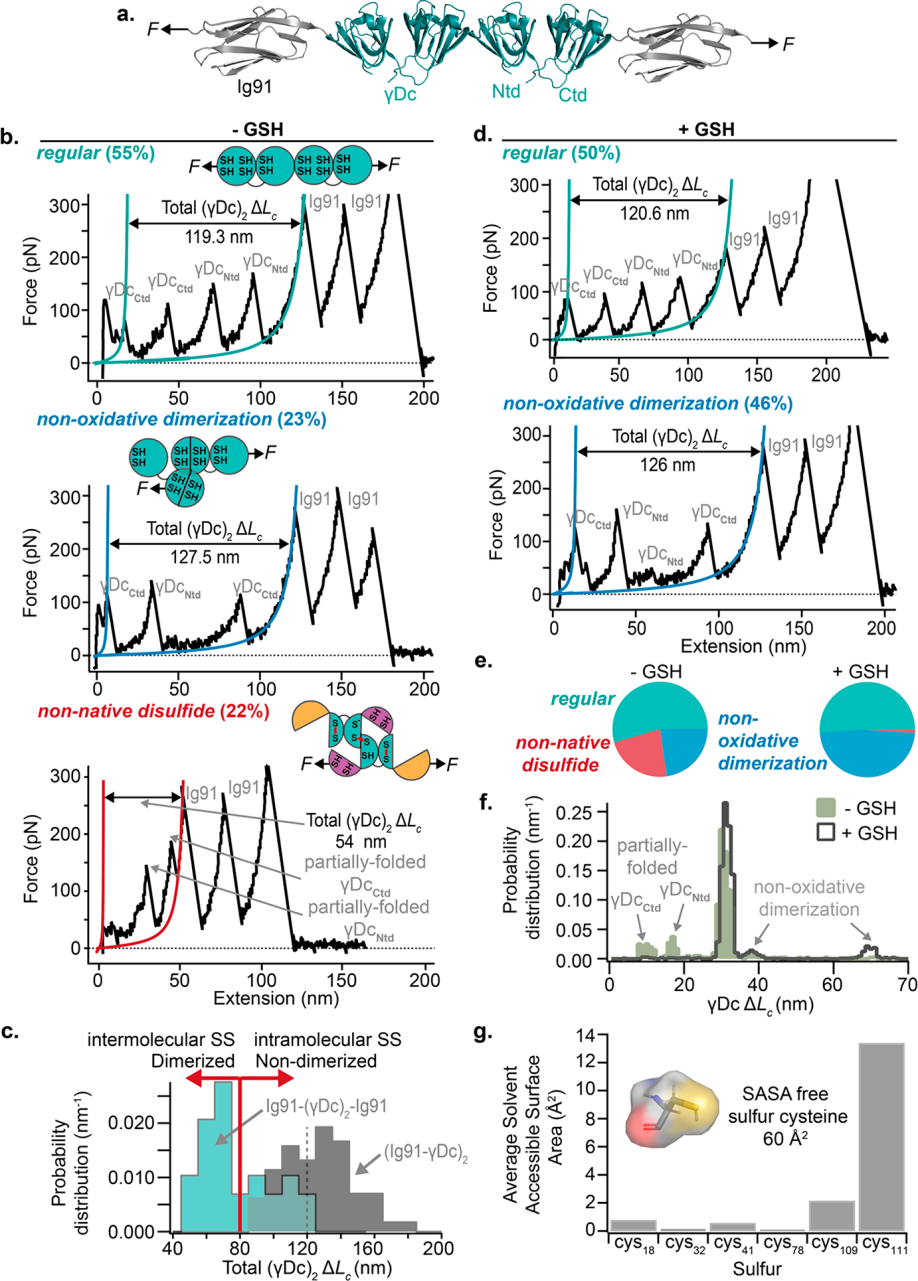
Capturing non-native intermolecular disulfide bridges in γDc. (a) Schematics of the engineered Ig91–(γDc)_2_–Ig91 polyprotein, containing two physically interacting γDc monomers. (b) Pulling on an individual Ig91–(γDc)_2_–Ig91 polyprotein at a constant velocity and in the absence of GSH results in three different unfolding phenotypes according to their length and peak position—*regular* (top), *non-oxidative dimerization* (middle), and *non-native disulfide* (bottom). The percentage values in brackets represent the frequency of each length phenotype. The *regular* unfolding trajectories (top) exhibit the initial unfolding of the two γDc monomers, releasing a total length of 120 nm. The *non-oxidative dimerization* length phenotype (middle) usually entails four γDc unfolding events with varying Δ*L*_c_ values and mechanical stabilities. The overall total Δ*L*_c_ (γDc)_2_ extension is larger than the *regular* phenotype by 7 nm. (c) Histogram comparing the total γDc (Δ*L*_c_)_2_ when pulling Ig91–(γDc_WT_)_2_–Ig91 (turquoise) and (Ig91−γDc_WT_)_2_ (gray) enabling to discriminate between (red vertical line) intramolecular (total γDc (Δ*L*_c_)_2_ > 80 nm) and intermolecular (total γDc (Δ*L*_c_)_2_ < 80 nm) non-native disulfide bridge formation in the absence of GSH. The *non-native disulfide* length phenotype (middle) typically displays a shorter total Δ*L*_c_ (γDc)_2_ extension and usually features partially folded conformations originating from either terminal. (d) Pulling on the Ig91–(γDc)_2_–Ig91 polyprotein in the presence of 1 mM of deprotonated GSH results in only two length γDc phenotypes: the *regular* (top) and the non-oxidative dimerization (bottom) unfolding pattern. (e) Pie charts showing the comparison between the different unfolding length phenotypes captured with and without GSH. (f) Histogram comparing the frequency of the γDc increment in contour-length when pulling the Ig91–(γDc)_2_–Ig91 polyprotein with and without GSH (*n* = 400 unfolding events without GSH and *n* = 1387 unfolding events with GSH). (g) Average sulfur solvent accessible surface area (SASA) of the 6 different cysteines comprised in γDc_WT_.

Having observed that γDc forms non-native intramolecular disulfide bonds, we questioned whether γDc can also establish intermolecular disulfide bonds with neighboring monomers. To this purpose, we built a polyprotein construct whereby two γDc monomers are physically connected to each other and flanked by the Ig91 marker proteins (Figure 3a). Pulling on the resulting Ig91–(γDc)_2_–Ig91 polyprotein resulted in a rich repertoire of unfolding trajectories, that could be classified into three mechanical phenotypes. In most cases (55%, Figure 3b, top), a *regular* (and expected) full-length unfolding trajectory consisting of four independent unfolding events of ∼30 nm (Supporting Figure 7) was observed. We also identified two distinct unexpected unfolding pathways that markedly departed from this *regular* behavior. First, around 23% of the trajectories exhibited a well-defined unfolding pattern (Figure 3b, middle), where the typical mechanical hierarchy is lost, exhibiting unfolding peaks of alternating higher and lower mechanical stabilities. This unfolding scenario is overall compatible with γDc dimerization through a domain swap mechanism via Ntd interchange that we described earlier.^37^ Importantly, the unfolding pattern does not exhibit any shortening in the (γDc)_2_ length, implying that domain swap-mediated dimerization maintains the native redox status of all 12 cysteines comprised in the two γDc monomers (Supporting Figure 8). The remaining 22% of the unfolding trajectories featured a significant decrease in the total (γDc)_2_ unfolding length (Figure 3b, bottom), which is likely to be underpinned by the formation of non-native disulfide bridges. Direct comparison of the measured total Δ*L*_c_(γDc)_2_ for the Ig91–(γDc)_2_–Ig91 polyprotein and the previously characterized (Ig91−γDc)_2_ construct enabled us to directly identify whether the formation of non-native disulfide bridges occurs within the same protein or between the two adjacent γDc monomers, Figure 3c. We conclude that, when the total Δ*L*_c_ (γDc)_2_ is shorter than 80 nm (light blue part of the histogram in Figure 3c), the two neighboring γDc monomers have dimerized through the formation of, at least, one non-native intermolecular disulfide bridge.

Examining the nanomechanical response of the same Ig91–(γDc)_2_–Ig91 polyprotein in the presence of reduced GSH (and TCEP, Supporting Figure 9) resulted in the absence of the shorter unfolding length phenotype, confirming that the formation of non-native disulfide bonds account for the shortening in (γDc)_2_ extension. Under these reducing conditions, the two other unfolding phenotypes, namely the *regular* (Figure 3d, top) and the non-oxidative dimerization pathway (Figure 3d, bottom) were observed in an almost equal probability (Figure 3e). As before, the histogram of γDc Δ*L*_c_ clearly shows that the population of partially folded conformations vanishes after exposure to reducing conditions, while the Δ*L*_c_ defining the domain swapped conformation increases its probability of occurrence (Figure 3f).

**Figure 4:**
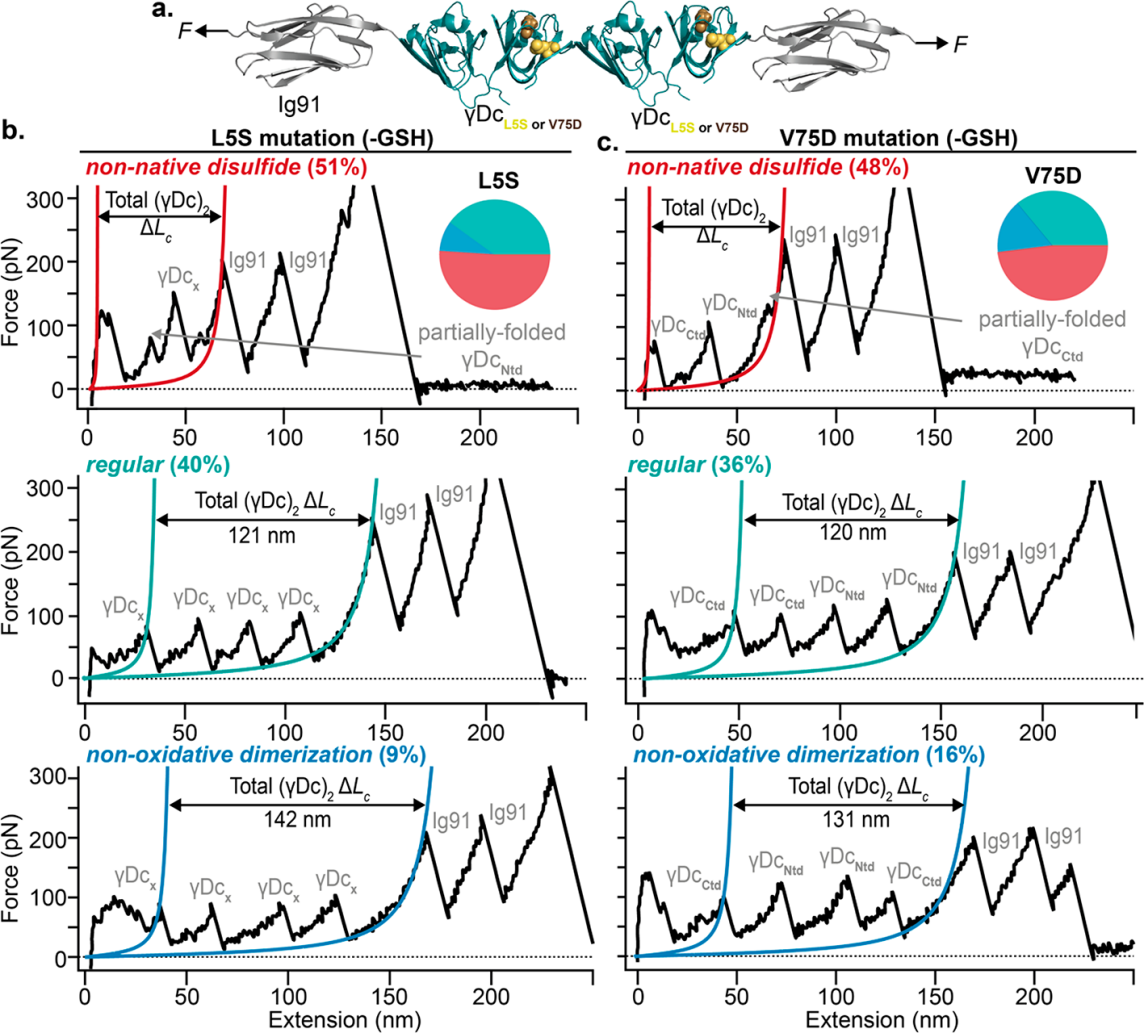
Single-point mutations enhance the formation of non-native intra- and intermolecular disulfide bridges. (a) Schematics of the engineered Ig91–(γDc_L5SorV75D_)_2_–Ig91 polyproteins. (b) Pulling on the polyprotein construct harboring the L5S congenital mutation in each γDc monomer results in unfolding trajectories featuring three different length phenotypes—*non-native disulfide* (top), *regular* (middle), and *non-oxidative dimerization* (bottom). (c) Pulling on a related single polyprotein construct harboring the V75D congenital mutation displays force–extension unfolding trajectories populating the three different length phenotypes—*non-native disulfide* (top), *regular* (middle), and *non-oxidative dimerization* (bottom).

The first obvious and unavoidable requirement for the formation of intermolecular disulfide bonds is that the involved cysteines from both neighboring proteins are surface-exposed. To evaluate the degree of sulfur exposure of the 6 different cysteines in each γDc monomer we conducted MD simulations, which demonstrated that only the sulfur in cys^111^ (in the Ctd) is partially exposed to the environment, while the other 5 cysteines are completely buried in the folded structure (Figure 3g). This finding suggests that, for the intermolecular disulfide bonds to occur, the individual γDc monomers need to undergo a conformational change (in the absence of force) that destabilizes the protein, ultimately resulting in cysteine exposure to the solvent. With this rationale in mind, we aimed to elucidate whether protein destabilization enhances non-native intermolecular disulfide formation. Given that 4 of the cysteines in γDc are present in the N-terminal, where, incidentally, most of the congenital cataract-point mutations cluster,^38,39^ we hypothesized that the introduction of single-point mutations in the Ntd could lead to destabilization, hence increasing the probability of cysteine exposure. We, first, used MD simulations to compare the sulfur solvent accessibility of the cysteines in the wild-type form with those in two pathogenic mutants, L5S and V75D,^40^ which overall did not show any significant change in the sulfur accessibility (Supporting Figure 10). We then constructed two different polyprotein constructs (Ig91–(γDc_L5S_)_2_–Ig91 and Ig91–(γDc_V75D_)_2_–Ig91) independently harboring each congenital cataract point mutation (Figure 4a). Pulling on the Ig91–(γDc_L5S_)_2_–Ig91 polyprotein in an oxidizing (PBS) environment (Figure 4b) revealed that the most prevalent pathway (50%) corresponded to the unfolding trajectories featuring a reduction in the overall (γDc)_2_ length, reminiscent of the formation of non-native disulfide bridges. The second most captured unfolding pathway (42%) followed the *regular* mechanical unfolding, and only 8% of the unfolding trajectories displayed the non-oxidative domain-swap dimerization unfolding pattern (Supporting Figure 11b). Noteworthy, for this L5S mutant, the mechanical stability of the Ntd is significantly decreased with respect to the wild-type protein, probably due to fact that the L5S mutation is located on the first β-strand of the Ntd (the protein’s mechanical clamp), making both protein terminals mechanically indistinguishable (Supporting Figure 12). Pulling on the Ig91–(γDc_V75D_)_2_–Ig91 protein (Figure 4c) gave rise to very similar results, whereby the predominant unfolding pathway (48%) comprises unfolding trajectories featuring a shorter (γDc_V75D_)_2_ unfolding length, followed by the *regular* (36%) and domain swapped (16%) unfolding phenotypes (Supporting Figure 11c). Following a similar approach to that followed in Figure 3c, the measured Δ*L*_c_ (γDc_X_)_2_ histogram reveals that, for both protein mutants, the proportion of intra- and intermolecular non-native disulfide formation is roughly the same (Supporting Figure 13). Combined, these experiments point toward an enticing scenario, where the lower thermodynamic stability of the Ntd domain of the protein mutants^38^ would enhance a conformational change that would expose previously buried cysteines, increasing the probability of non-native disulfide bond formation—a situation that becomes prevalent.

## Discussion

Whether beneficial or deleterious for the cell,^41,42^ the detection of non-native, redox-regulated disulfide bridges has been particularly challenging. To date, most of the relevant studies use refined proteomics methods to tag^43^ and capture these low-probability covalent bonds. Their highly dynamic nature, their fast reactivity, and their intimate relationship with the dynamically evolving protein structure necessitates the development of complementary experimental approaches.

**Figure 5:**
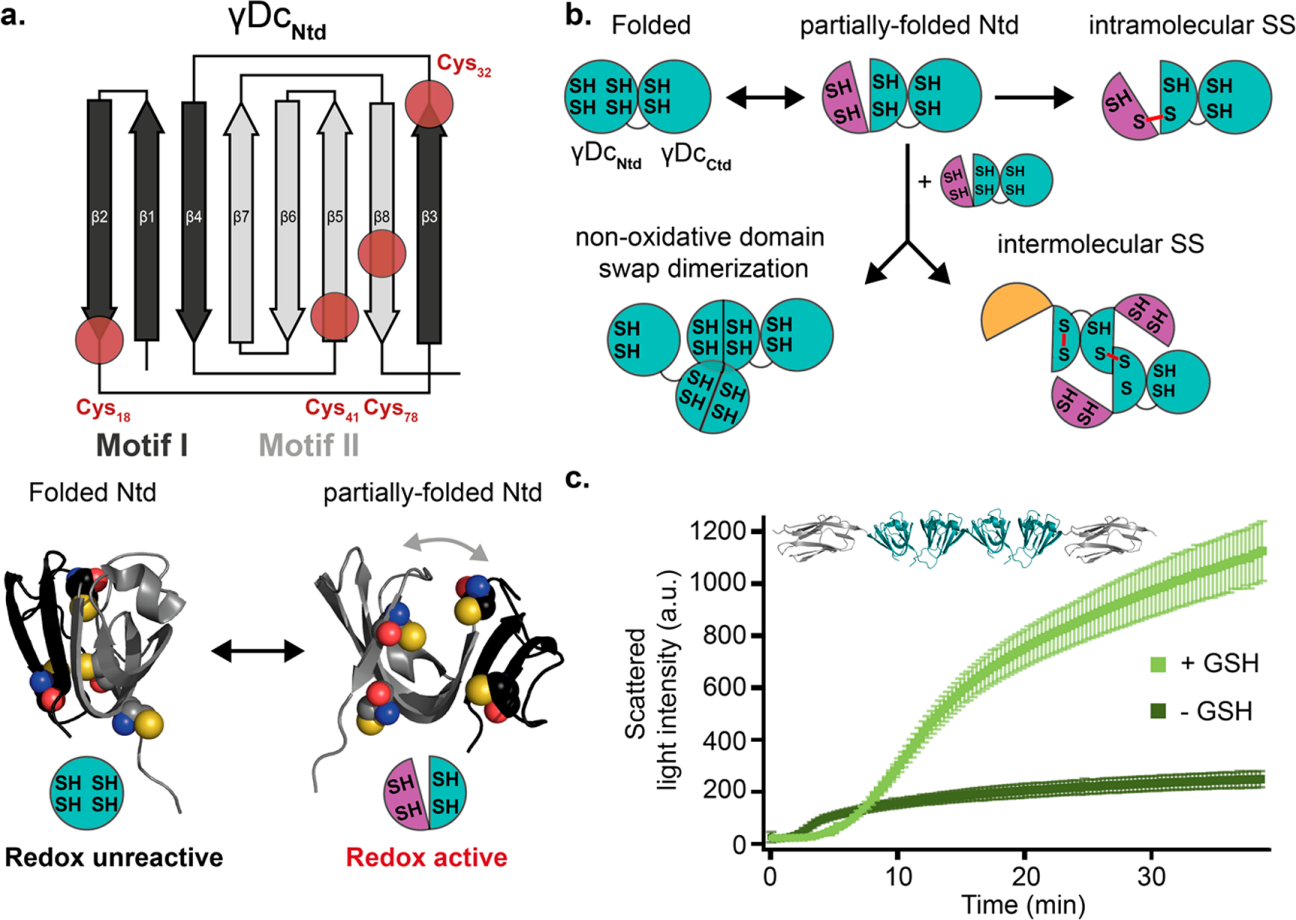
Spontaneous Ntd conformational switch reveals cryptic cysteines and enables their reactivity. (a) Plausible structural change spontaneously undergone by γDc Ntd, involving a partially folded conformation characterized by the hinge-like detachment of motif I from motif II, resulting in the concomitant solvent-exposure of at least three cysteines that become suddenly reactive. (b) Schematic mechanistic representation of the cysteine-redox regulated conformational diversity encountered in γDc. (c) Aggregation kinetics of the Ig91–(γDc)_2_–Ig91 polyprotein in the presence and absence of GSH, measured by light scattering at 60 °C GSH (*n* = 4 for each condition, error bars s.e.m.).

Here, we use single-molecule force spectroscopy combined with protein engineering as a tool to capture non-native disulfide bonds by using molecular extensibility as their structural fingerprint. This methodology could be applied to virtually any protein, regardless of whether its physiological function is mechanical or not. As a proof-of-principle, we studied the redox-regulation of human γD-crystallin (γDc), the misfolding of which is directly related to eye’s cataract. Our single-molecule experiments reveal the presence of both inter- and intramolecular non-native disulfide bonds in γDc. Pulling on the (Ig91−γDc)_2_ polyprotein revealed the presence of non-native intramolecular disulfide bonds, directly linked to the existence of partially folded conformations found in both γDc_Ctd_ and γDc_Ntd_ domains. The γDc_Ctd_ has only two cysteines (cys^109^ and cys^111^), a priori limiting the possible non-native disulfide bonds to the one (cys^109^–cys^111^) proposed by Serebryany et al.^44^ However, the predominant intermediate conformation in γDc_Ctd_ is characterized by Δ*L*_c_ ∼9 nm, which would correspond to a “trapped length” much larger than that corresponding to the (cys^109^–cys^111^) disulfide (Supporting Figure 14). To rationalize this, we conducted MD simulations, showing that the formation of the non-native disulfide bridge in the γDc_Ctd_ can indeed induce a non-native partially folded protein conformation (Supporting Figure 15) compatible with the Δ*L*_c_ ∼9 nm that we observe. This change in γDc_Ctd_ conformation enforced by the formation of the non-native disulfide bridge is also likely accountable for the higher mechanical stability of the partially folded conformation (∼150 pN) when compared to the folded γDc_Ctd_ structure (∼100 pN). A closer look into the γDc_Ntd_ structure shows that the 4 cysteines are distributed evenly among the two Greek key motifs. Given that γDc_Ntd_ shows a partially folded conformation characterized by a Δ*L*_c_ ∼15 nm, a plausible scenario entails visiting a partially folded conformation whereby motif I is unfolded, while motif II remains folded (Supporting Figure 16). This partially folded conformation completely exposes three cysteines (cys^18^, cys^32^, and cys^41^), which could explain the presence of both intramolecular and intermolecular disulfide bonds, the latter being uncovered when pulling on the Ig91–(γDc)_2_–Ig91 polyprotein. Given that our molecular dynamics simulations revealed that, in the folded form, only one cysteine in the Ctd is solvent-accessible, it is highly plausible that a conformational change (which we speculate involves the spontaneous unfolding of half of the Ntd) that exposes cryptic cysteines is a necessary step prior to disulfide bond formation. Interestingly, the partial unfolding of the Ntd motif I was suggested to swap with a neighboring γDc domain.^37^ We, therefore, propose a plausible global scenario able to explain the three observed types of non-native conformations; if γDc remains in the native state, it exhibits a *regular* pattern of unfolding. However, once the protein undergoes a conformational change involving a “hinge” that opens Ntd motif I (Figure 5a), then two competing situations emerge. Either the protein swaps with a neighboring domain (eliciting the domain swapped conformations), or it forms non-native disulfide bonds (either intra- or intermolecular), marked by the “shorter” unfolding phenotype (Figure 5b).

An intrinsic limitation of our approach is that, given the number of cysteines present in each γDc monomer, and hence of possible disulfide cross-links, we do not have the resolution to identify which cysteine pairs are involved in each particular non-native disulfide bond formed between adjacent domains. The intrinsic design of the polyprotein chain is also likely to restrict the number of possible intermolecular disulfide bonds, if compared to the much larger number of available conformations when the interacting monomers are in solution. Conversely, the polyprotein approach has the advantage of offering unprecedented control over the onset of protein misfolding, occurring at the dimer level. Functionally, it remains unknown what is the relationship between non-native disulfides and domain swapped structures. In the presence of GSH (Figure 3e) the population of “non-native disulfide trajectories” disappears, while the domain swapped population grows by essentially the same amount (from ∼20% to ∼50%), hence suggesting that these two conformations are in dynamic competition. As a first step toward relating these single-molecule observations to bulk aggregation measurements, we measured the kinetics of light scattering of the Ig91–(γDc)_2_–Ig91 polyprotein in the absence and presence of reduced GSH (Figure 5c). Our results showed a drastic increase in the aggregation of the Ig91–(γDc)_2_–Ig91 polyprotein in the presence of GSH, strongly suggesting that the non-native disulfide bonds that we measured at the single-molecule level act as a safety mechanism that prevents protein aggregation, probably by limiting the misfolded, domain swapped conformation that leads to protein aggregation. From the physiological perspective, given that the eye lens are separate from the blood vessels and lack protein regeneration machinery, and that its glutathione concentration unavoidably diminishes over time,^45^ it is tempting to speculate that the amount of non-native disulfide bonds, which is one of the key initial steps toward developing cataracts,^46^ will increase over the life-span of the individual.

Altogether, our experiments demonstrate the power of single-molecule spectroscopy to capture non-native disulfide bonds, both intra- and also intermolecular, by using the protein extension as a conformational reporter. In the specific case of γDc, non-native disulfide formation is likely to follow from a stochastic yet well-defined conformational excursion of the Ntd to a partially folded conformation that exposes previously cryptic cysteines to the solvent, hence rendering them reactive. We anticipate that our combined experimental platform, encompassing protein engineering, single-molecule force spectroscopy experiments, MD simulations and classical biochemistry techniques can help uncover the dynamic nature of non-native protein disulfide bonds, of wide occurrence in nature.

## Supporting Information Available

Details of polyprotein engineering, single-molecule AFM setup and data analysis, light-scattering experiments, molecular dynamics simulations, and additional data sets (PDF)
