# Supplementary material for: A Single-Molecule Strategy to Capture Non-native Intramolecular and Intermolecular Protein Disulfide Bridges
Source: Nano Lett. 2022 May 12;22(10):3922–30. doi: 10.1021/acs.nanolett.2c00043 (PMC9136921; doi:10.1021/acs.nanolett.2c00043)
Supplement: Supplementary file 1 — nl2c00043_si_001.pdf [file nl2c00043_si_001.pdf]

Supplementary Online Information for:

A single-molecule strategy to capture non-native intramolecular and intermolecular protein disulfide bridges

Marc Mora[1,2¶], Stephanie Board[1,2], Olivier Languin-Cattoën[3], Laura Masino[4], Guillaume Stirnemann[3], Sergi Garcia-Manyes[1,2¶]

[1]Department of Physics, Randall Centre for Cell and Molecular Biophysics and London Centre for Nanotechnology and London Centre for Nanotechnology, King's College London, Strand, WC2R 2LS London, United Kingdom.
[2]Single Molecule Mechanobiology Laboratory, The Francis Crick Institute, 1 Midland Road, London NW1 1AT, London, United Kingdom.
[3]CNRS Laboratoire de Biochimie Théorique, Institut de Biologie Physico-Chimique, Univ. Paris Diderot, Sorbonne Paris Cité, PSL Research University, 13 rue Pierre et Marie Curie, 75005 Paris, France
[4]Structural Biology Science Technology Platform, The Francis Crick Institute, 1 Midland Road, London NW1 1AT, United Kingdom.

[¶]Corresponding authors: marc.mora_hortal@kcl.ac.uk, sergi.garcia-manyes@kcl.ac.uk

Materials and methods

Protein engineering

The (Ig91- γDc_{Ntd})$_4$, (Ig91- γDc_{Ctd})$_4$ (Ig91- γDc)$_2$, (Ig91- γDc_2-Ig91), (Ig91- $\gamma Dc_{(L5S)2}$-Ig91) and (Ig91- $\gamma Dc_{(V75D)2}$-Ig91) polyproteins were subcloned using the BamHI, BglII and KpnI restriction sites. The L5S and V75D mutations were created by site directed mutagenesis. All polyprotein constructs were cloned into the pQE80L (Qiagen) expression vector, and transformed into the BLR(DE3) *Escherichia coli* expression strain (Novagen) and T7 Express competent E. Coli (NEB). Cells were grown in LB broth supplemented with 100 μg/mL of ampicillin at 37 ^{o}C. After reaching an OD_{600} of $\sim$0.6, cell cultures were induced with Isopropyl β-D-1-thiogalactopyranoside (1 mM) and incubated overnight at 20 ^{o}C. After harvesting, cells were disrupted using a French Press was performed. The polyproteins from lysate were purified by metal affinity chromatography on Talon resin (Clontech) followed by gel-filtration using a Superdex 200 increase 10/300 GL column (GE Biosciences).

Single-molecule force-spectroscopy experiments

Single-molecule force-extension using Atomic Force Microscopy (AFM) were conducted at room temperature using both a home-made set-up[1] and a commercial Luigs and Neumann force spectrometer as described previously[2]. Briefly, the sample was prepared by depositing 0.3-2.5 μL of fresh protein (at a concentration of 0.5-5 mg ml^{-1}) in PBS, TCEP or GSH solution onto freshly evaporated and plasma cleaned gold cover slides. Prior to each experiment, the AFM cantilever (Si_3N_4 Bruker MLCT-AUHW) was individually calibrated using the equipartition theorem, giving rise to a typical spring constant of $\sim$12-22 pN nm^{-1}. Individual polyproteins were pulled by first pushing the cantilever onto the surface (from 200 to 2200 pN) to enhance the non-specific interaction of the polyprotein construct onto the cantilever. The piezoelectric actuator was then retracted at a constant velocity of 400 nm s^{-1}. When using reducing agents in the experiments (pH=7.5), GSH (Sigma Aldrich, 98%) was kept at a deprotonated concentration of 1 mM. TCEP solutions used a total concentration of 4 mM. All buffers were diluted into a sodium phosphate buffer solution, precisely, 50 mM sodium phosphate (Na_2HPO_4 and NaH_2PO_4) and 150 mM of NaCl. The final pH of each solution was adjusted by adding the required amounts of NaOH (1.5 M). Freshly prepared solutions were filtered through a 0.2 μm membrane before each experiment.

Data analysis

All measured data were recorded and analysed by using a custom written software in Igor Pro (Wavemetrics). For the experiments conducted using (Ig91- γDc)$_2$, (Ig91- γDc_2-Ig91) and (Ig91- $\gamma Dc_{(V75D/L5S)2}$-Ig91), only polyproteins featuring the unfolding of the two Ig91 events were considered for analysis. For the experiments conducted using (Ig91- γDc_{Ntd})$_4$, (Ig91- γDc_{Ctd})$_4$ polyproteins, only force-extension unfolding trajectories featuring at least three Ig91 unfolding events were considered for analysis.

Light-scattering experiments

Polyprotein constructs were first thermally equilibrated at 60 °C for 10 minutes (at a $\sim$90 μM concentration). Then the oligomerization was monitored by measuring scattered light at 60 °C using a JASCO FP-8300 Spectrofluorometer, with excitation and emission wavelengths of 350 nm, and

excitation and emissions bandwidths of 2.5 and 0 nm, respectively. When using a reducing buffer, the concentration of deprotonated GSH was kept at 0.5 mM.

Molecular dynamics simulations

System preparation

All systems for Molecular Dynamics simulations were prepared from the structure of human γD-Crystallin (PDB: 1hk0[3]), using either the full protein (res. G^1-S^{174}) or the isolated C-terminal domain (S^{87}-S^{174}). For the full protein, mutations L5S and V75D were engineered in Chimera[4].

Topologies were prepared with GROMACS 2018.4[5] using AMBER99SB-disp[6] force field. The proteins were solvated in a dodecahedral box ensuring at least a 1.5 nm distance between the solute and the box sides. The systems were equilibrated at 300 K using steepest gradient descent followed by a 100 ps NPT simulation with restraints on heavy atoms.

For the system with the non-native C^{109}-C^{111} disulfide bond, a short 400 ps steered MD simulation was used after equilibration to bring the sulfur atoms into close distance. A harmonic restraint at the crystal structure distance $d_{S\text{-}S}$ = 6.74 Å was progressively turned on (0-100 ps) by linearly increasing the harmonic constant from 0 to κ = 500000 kJ/mol/nm². Then, its center was gradually moved to a final $d_{S\text{-}S}$ = 2.05 Å (100 ps-400 ps). The final structure was used as a starting point of the C-terminal domain containing a C^{109}-C^{111} disulfide bond, which was then equilibrated following the procedure described above.

Conformational sampling

MD simulations were carried out using GROMACS 2018.4 patched with PLUMED 2.5[7] to investigate the conformational landscape of γDc Ntd and Ctd domains. We used Replica Exchange with Solute Scaling (REST2)[8] in order to enhance sampling. REST2 is similar to traditional temperature replica exchange (parallel tempering), but instead of changing the system's temperature, modified potential energy functions are used:

$$U_i = \left(\frac{T_0}{T_i}\right) U_{pp} + \sqrt{\left(\frac{T_0}{T_i}\right)} U_{ps} + U_{ss}$$

Where U_i is the potential energy of the i-th replica, U_{pp}, U_{ps} and U_{ss} are the protein-protein, protein-solvent and solvent-solvent interactions, T_0 is the temperature of the base (unscaled) replica, and T_i is the desired effective temperature at the i-th replica. N replicas following a geometric range between effective temperatures 300 K and 600 K were used (N = 24 for the full protein and N = 16 for Ctd). Exchanges between neighboring replicas were attempted every 1 ps alternating between even and odd pairs and accepted using the Metropolis-Hastings criterion. All simulations used a 2 fs timestep, a force-switch 1 Å to 1.2 Å cutoff for van der Waals interactions, a 1.2 Å cutoff for short-range Coulomb interactions combined with the particle mesh Ewald method for long-range electrostatics. The physical temperature was set to 300 K using two separate v-rescale thermostats for the protein and the solvent, with a 0.1 ps coupling time constant. Pressure was set to 1 bar using a Parrinello-Rahman barostat, with a 2 ps coupling constant.

REST2 simulations for each system were run for 300 ns and configurations were saved every 100 ps. The first 100 ns were discarded as equilibration. The last 200 ns of the unscaled replica (T=300 K) are used for analysis.

SASA calculations

The solvent accessible surface area of the sulfur atoms were computed using VMD's[9] native tools. A solvent radius of 1.4 Å was used. Distributions and averages were analyzed in Python.

Clustering

The clusterings of Ctd SH SH and Ctd S-S were done with GROMACS gmx cluster tool, using pairwise Cα RMSD as a distance metric and the linkage algorithm with a RMSD cutoff of 2 Å. A single cluster was found for the SH SH trajectory. Meanwhile, 5 were found for the S-S one, among which 2 accounted for 99.4% of the data. The first (82.7%) corresponded to the native structure and the second (16.7%) to the partially disrupted intermediate shown in Sup. Fig. 13b.

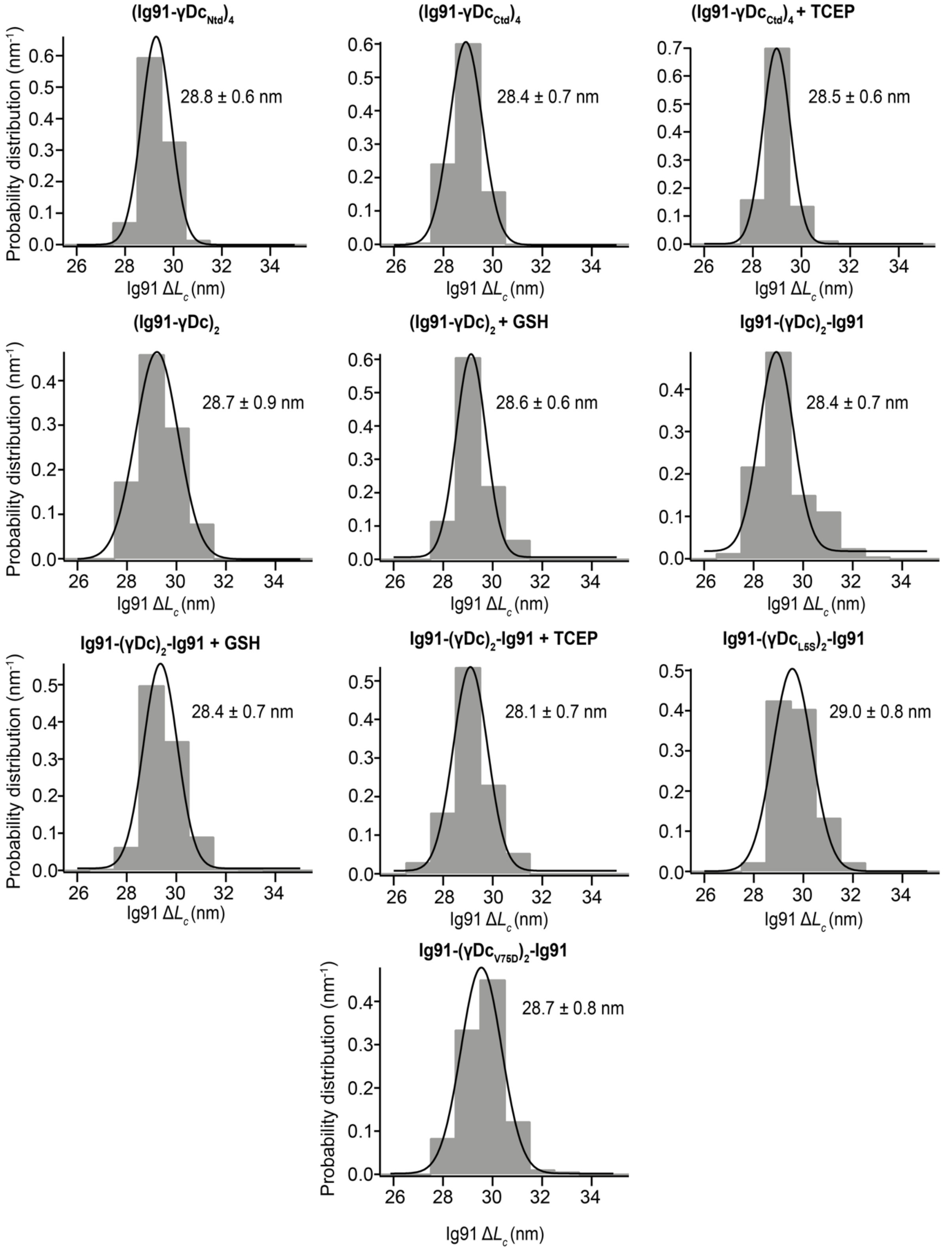

Supplementary figure 1. Ig91 increment in contour length analysis for all mechanically studied polyprotein constructs and at the different redox conditions employed (increment in contour length $\pm$ s.e.).

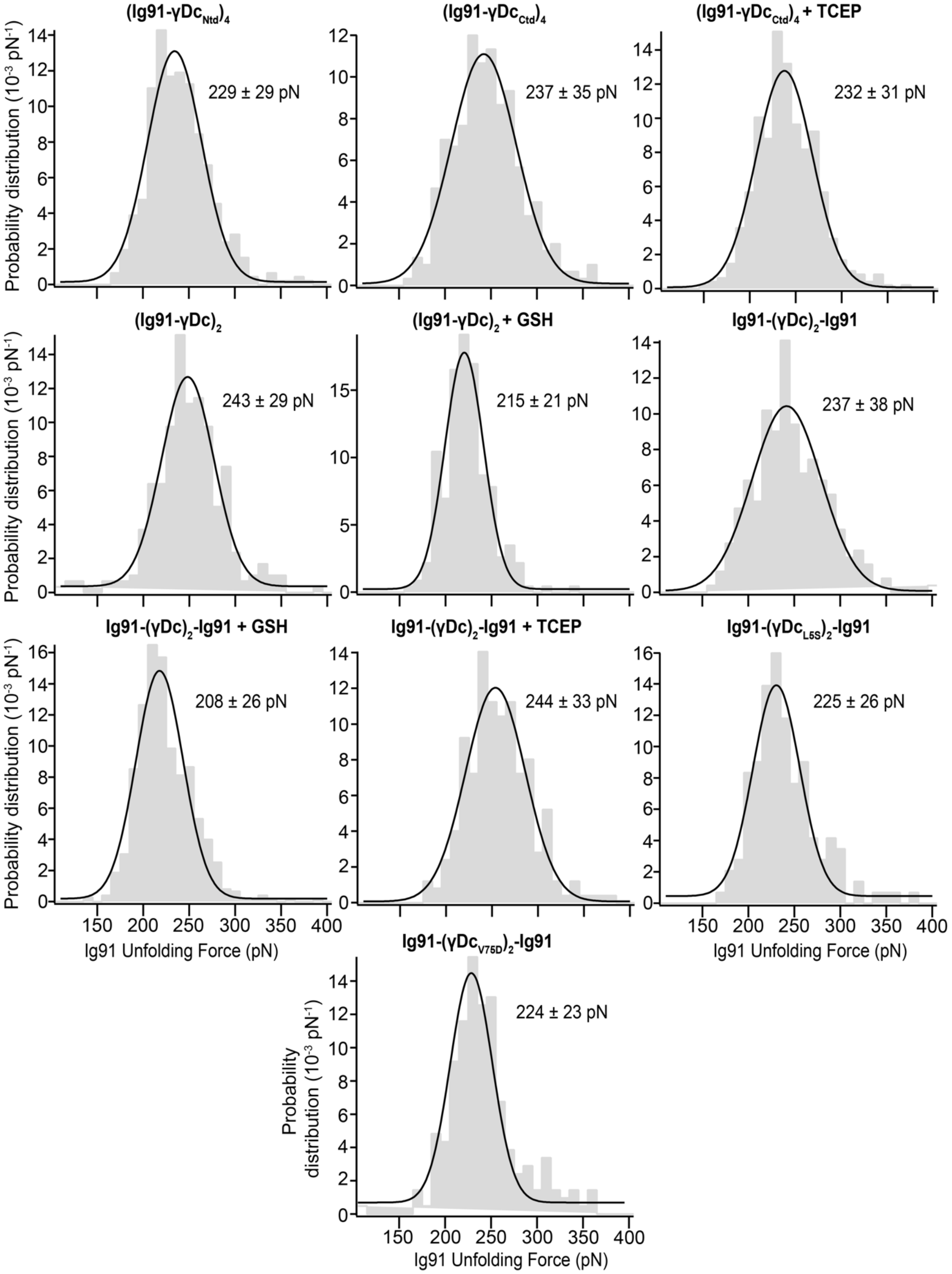

Supplementary figure 2. Ig91 unfolding force analysis for all the mechanically studied polyprotein constructs and at the different redox conditions employed (unfolding force ± s.e.).

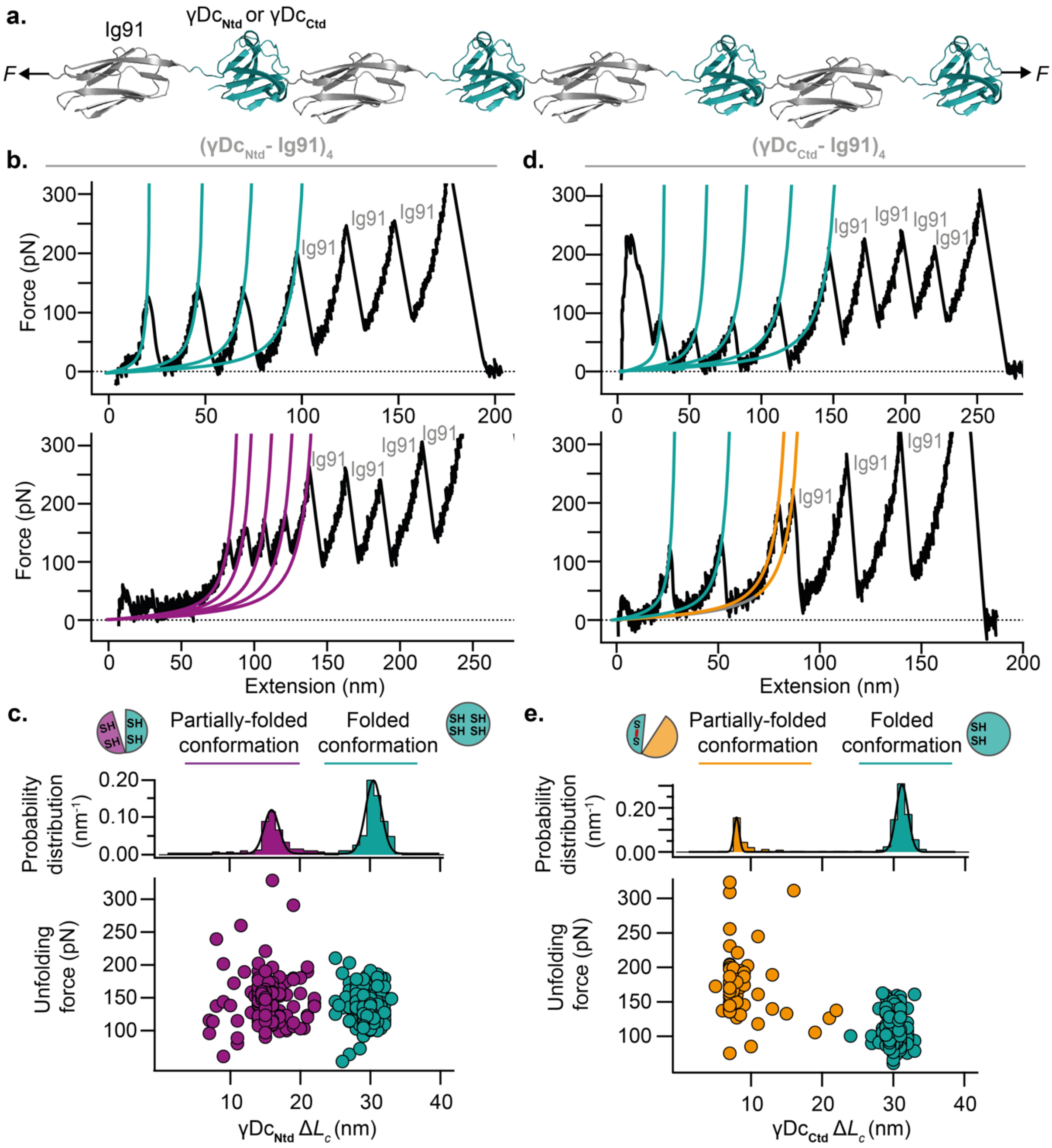

Supplementary figure 3. The mechanical unfolding trajectories of each independent γDc domain reveal two distinct unfolding events. a. Schematics of the engineered (Ig91- γDc$_{\mathrm{Ntd\ or\ Ctd}}$)$_4$ polyprotein. **b.** Two representative unfolding trajectories of an individual (Ig91- γDc$_{\mathrm{Ntd}}$)$_4$ polyprotein under force-extension conditions. The most frequent type of unfolding trajectory (top) features unfolding peaks of different mechanical stabilities; the lower mechanical stability peaks corresponding to the unfolding of the Ntd (turquoise WLC fits) and the higher mechanical stability peaks to the unfolding of the Ig91 marker (grey WLC fits). (Bottom) Lower prpbability unfolding trajectory, whereby all the 4 Ntds are in a partially-folded conformation (purple WLC fits), featuring a smaller increment of contour length (~15 nm) than the folded protein (~30 nm), followed by the unfolding of the Ig91 domains (grey WLC fits). **c.** A scatterplot displaying the relationship between the γDc$_{\mathrm{Ntd}}$ ΔL_c and the unfolding force (bottom). Purple circles correspond to unfolding of partially folded events and turquoise circles correspond to the unfolding of the natively folded Ntd conformation. The top histogram shows the frequency of occurrence of each γDc$_{\mathrm{Ntd}}$ ΔL_c event (*n*= 396 unfolding events). F_{Ntd}=131±15 pN and

$\Delta L_{c,Ntd}$=29.0±1.1 nm (s.e.). **d.** Two representative (Ig91- γDc$_{Ctd}$)$_4$ unfolding trajectories. In the most frequent scenario (top), the unfolding trajectories display four initial events corresponding to the unfolding of each Ctd (turquoise WLC fits) followed by four higher mechanical stability peaks corresponding to the unfolding of the Ig91 markers (grey WLC fits). The bottom unfolding trajectory exemplifies a less-frequent unfolding phenotype whereby one of the Ctds is in a partially-folded conformation (orange WLC fits), featuring a significantly shorter increment of contour length (~9 nm) than the native Ctd structure (~30 nm), followed by the unfolding of the Ig91 domains (grey WLC fits). **e.** A scatterplot (bottom) displaying the γDc$_{Ctd}$ ΔL_c — Force relationship whereby the orange circles correspond to the unfolding of the partially folded events and the turquoise circles, to the unfolding of the natively folded Ctd conformation. The top histogram shows the frequency of occurrence of each γDc$_{Ctd}$ ΔL_c event (*n*= 252 unfolding events). F_{Ntd}=100±19 pN and $\Delta L_{c,Ntd}$=29.7±1.0 nm (s.e.)

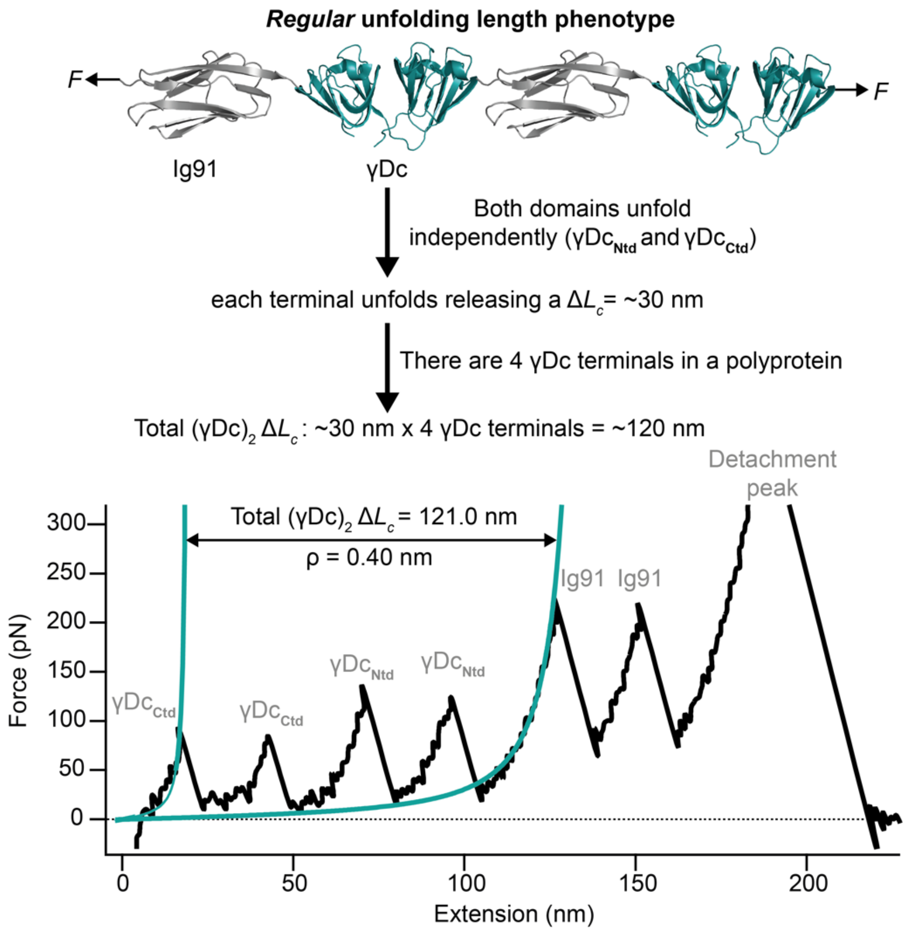

Supplementary figure 4. ***Regular*** **(Ig91-γDc)$_2$ unfolding trajectory.** Pulling on a polyprotein containing two γDc domains separated by two Ig91 protein domains typically unfolds releasing a total ΔL_c ~120 nm (corresponding to the independent unfolding of the four γDc domains) followed by the unfolding of the two Ig91 marker proteins. The γDc domains unfold independently and can be discriminated by the unfolding force (yet not by their ΔL_c). The γDc_{Ctd} exhibits a lower unfolding force (~100 pN) than the γDc_{Ntd} (~140 pN).

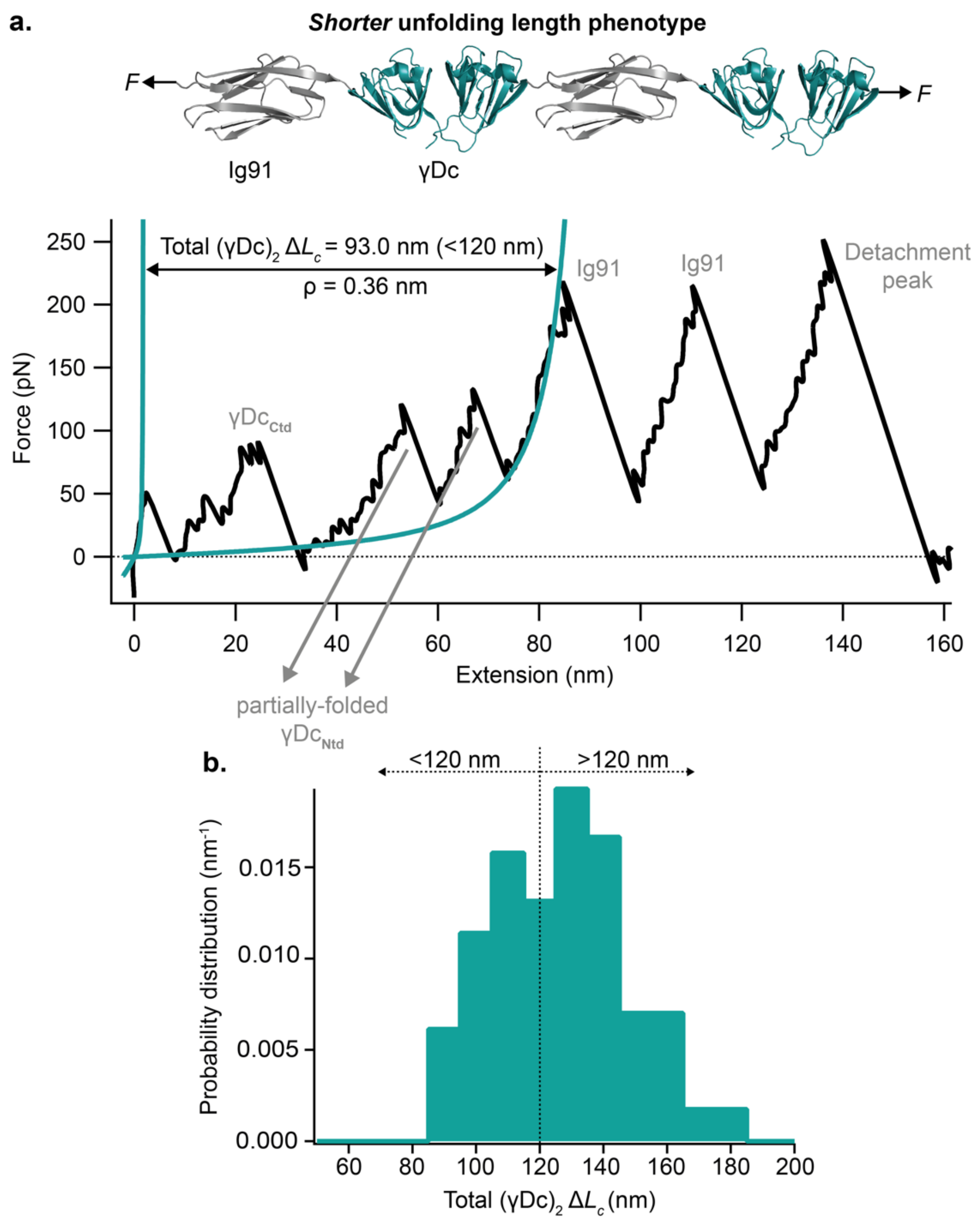

Supplementary figure 5. *Shorter* length (Ig91-γDc)$_2$unfolding trajectory. a. In some instances, pulling on (Ig91-γDc)$_2$ results in an initial extension shorter than that expected when fully unfolding and extending the two γDc domains (expected ~120 nm). This shortening is due to the formation of intramolecular non-native disulfide bridges within each γDc monomer. **b.** Histogram of the total (γDc)$_2$ ΔL_c when pulling (Ig91-γDc)$_2$ in the absence of a reducing agent (*n*=114 unfolding traces that contain the full two Ig91 unfolding fingerprint), showing that ~40% of the trajectories feature an intramolecular non-native disulfide bond.

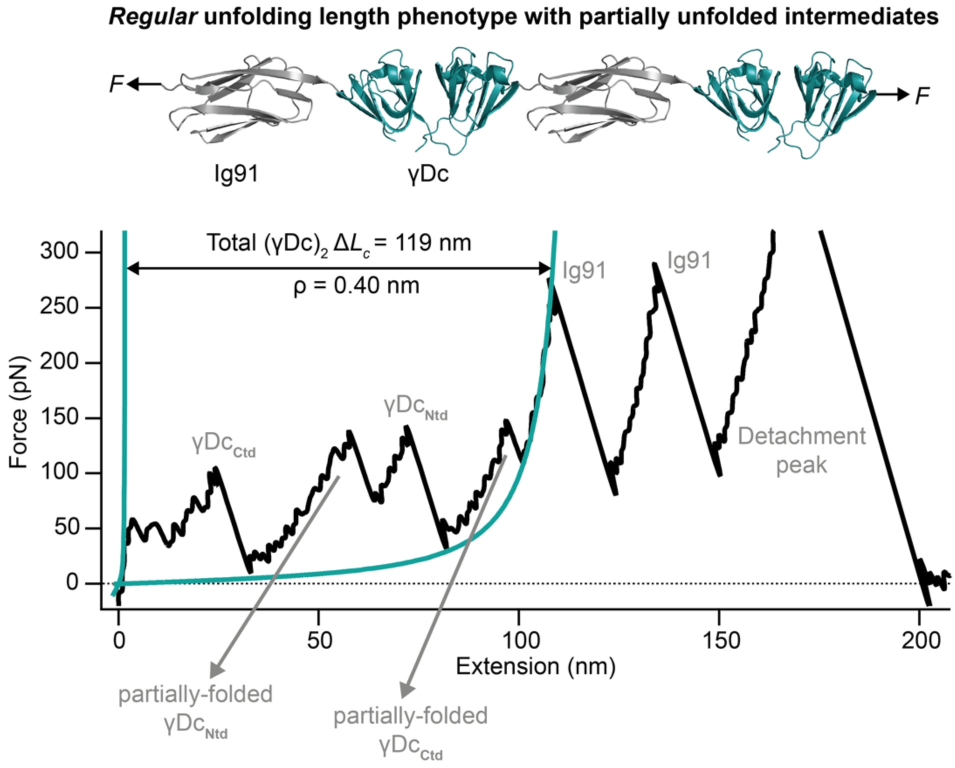

Supplementary figure 6. (Ig91-γDc)$_2$ unfolding trajectories displaying partially folded conformations yet the total length remains invariable ∼120 nm).

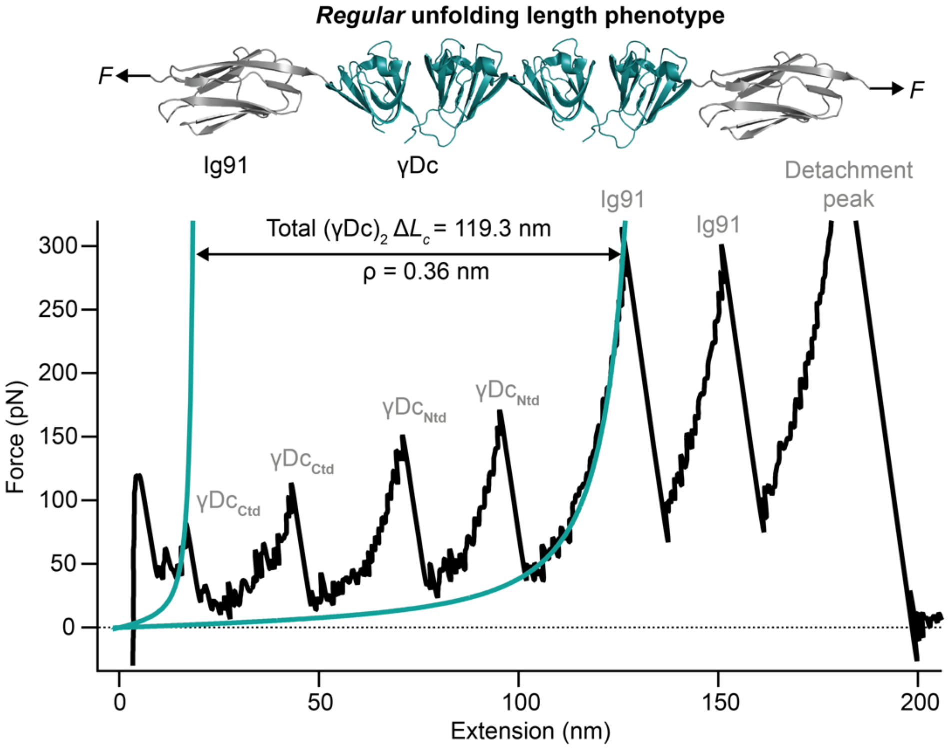

Supplementary figure 7. *Regular* Ig91-(γDc)$_2$-Ig91 unfolding trajectory. Representative (of the most common) force vs extension Ig91-(γDc)$_2$-Ig91 unfolding trajectory in the absence of a reducing agent, showing the initial unfolding of the two Ctds, followed by the unfolding of the two Ntds. Consequently, the total increase in contour length corresponding to the unfolding of both γDc domains is (γDc)$_2$ ΔL_c $\sim$120 nm.

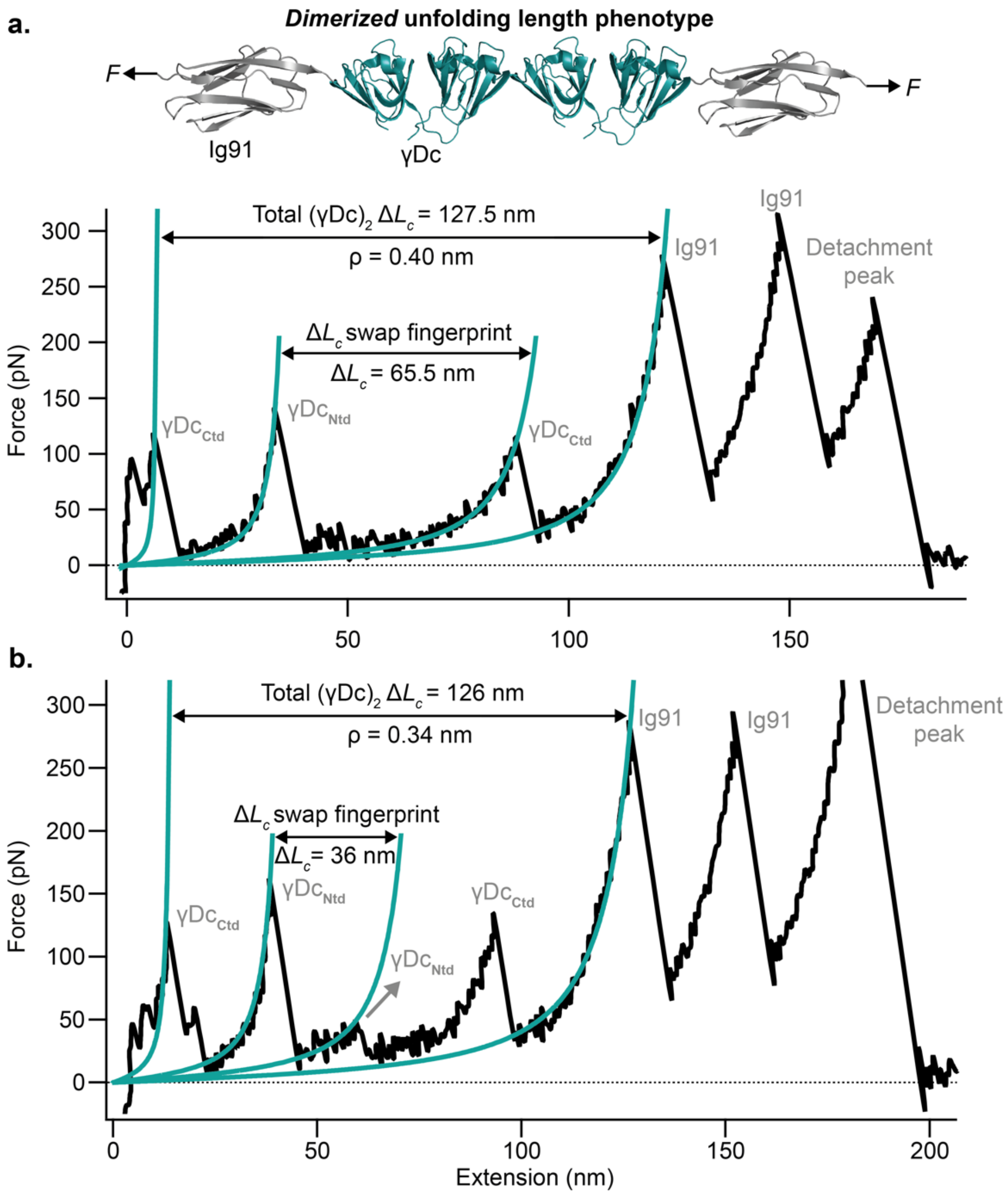

Supplementary figure 8. Unfolding trajectories of an individual Ig91-(γDc$_{WT}$)$_2$-Ig91 polyprotein fingerprint a non-oxidative dimerized (through domain-swap) conformation. Two representative Ig91-(γDc$_{WT}$)$_2$-Ig91 unfolding trajectories revealing a domain swapped conformation hallmarked by a slightly larger total (γDc)$_2$ ΔL_c (~127 nm) than the *regular* case (~120 nm). The small increment in the total γDc (ΔL_c)$_2$ is due to the structural rearrangement of the two Ntd when swapped. When the two Ntd are exchanged in a 3D-swapped conformation, they behave as a force-bearing element that prevents one of the Ctd from being exposed to mechanical force. In turn, the linker amino acids between the two proteins, as well as the natural amino acid linker between the Ntd and the Ctd (which are usually not contributing to the ΔL_c as they are unstructured) are released adding an extra length when mechanically disrupting the Ntd swapped conformation. This swapped conformation is also responsible for the disruption of the mechanical hierarchy expected when unfolding polyproteins. There are two specific 'mechanical fingerprints' that clearly single out those domain swapped (non-oxidative) conformations, namely **a.** one of the two interchanged Ntds does not almost display any mechanical resistance or **b**. significantly lower than expected (<~140 pN).

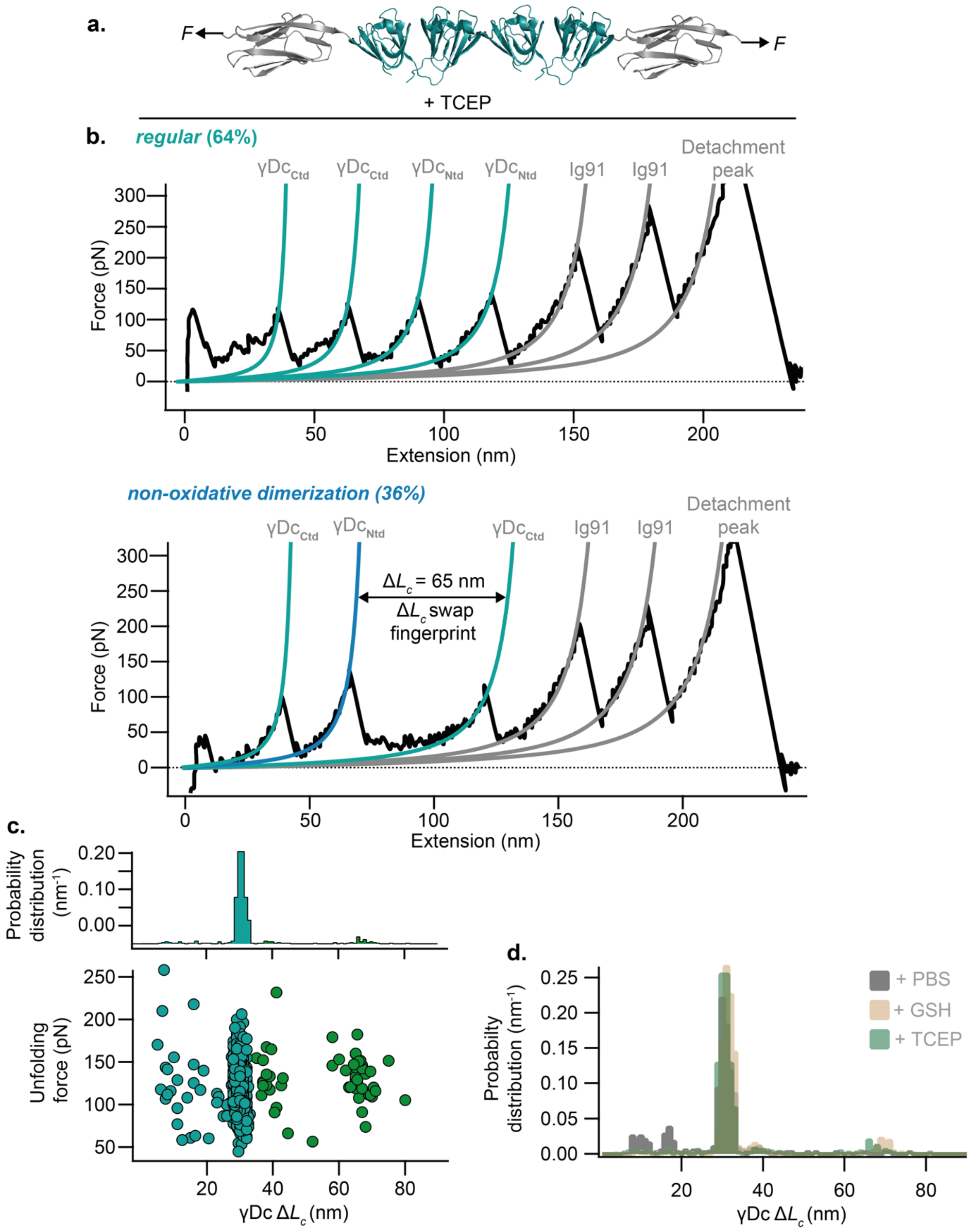

Supplementary figure 9. The redox-switch in the dimerization of γDc using GSH is recapitulated with TCEP. a. Schematics of the engineered polyprotein composed of two connected γDc monomers flanked by two Ig91 monomers in the presence of 4 mM of TCEP. **b.** Pulling the Ig91-(γDc_{WT})$_2$ polyprotein in the presence of TCEP results in two different length/mechanical phenotypes – *regular and dimerized* (top and bottom, respectively). The percentage values in brackets represent the frequency of occurrence of each phenotype. **c.** Scatterplot displaying the relationship between the γDc_{Ctd} increment in contour length and the unfolding force (bottom), alongside the related frequency of the unfolding events (top) in the presence of TCEP (*n*=523 unfolding events). **d.** γDc_{Ctd} ΔL_c

histogram related to Ig91-($\gamma Dc_{WT})_2$ unfolding in the presence of TCEP (light green), GSH (cream) and in the absence of reducing agent (grey).

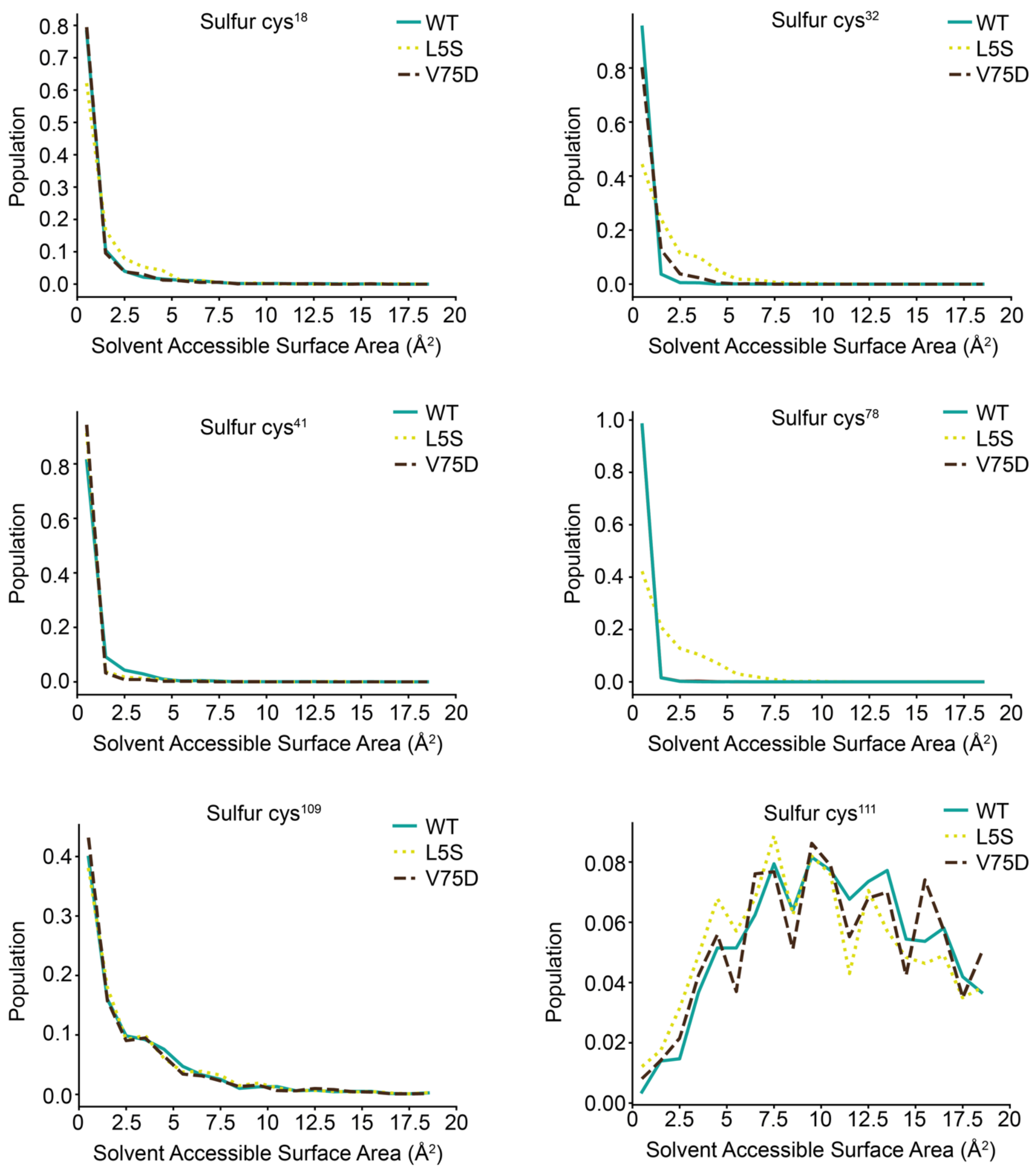

Supplementary figure 10. γDc cysteine$_{WT,\ L5S\ and\ V75D}$ sulfur solvent accessibility measurements. Comparison of the average Sulfur Solvent Accessible Surface (SASA) distributions of the 6 cysteines in the γDc wild type form and upon introduction of single-point mutations, L5S and V75D.

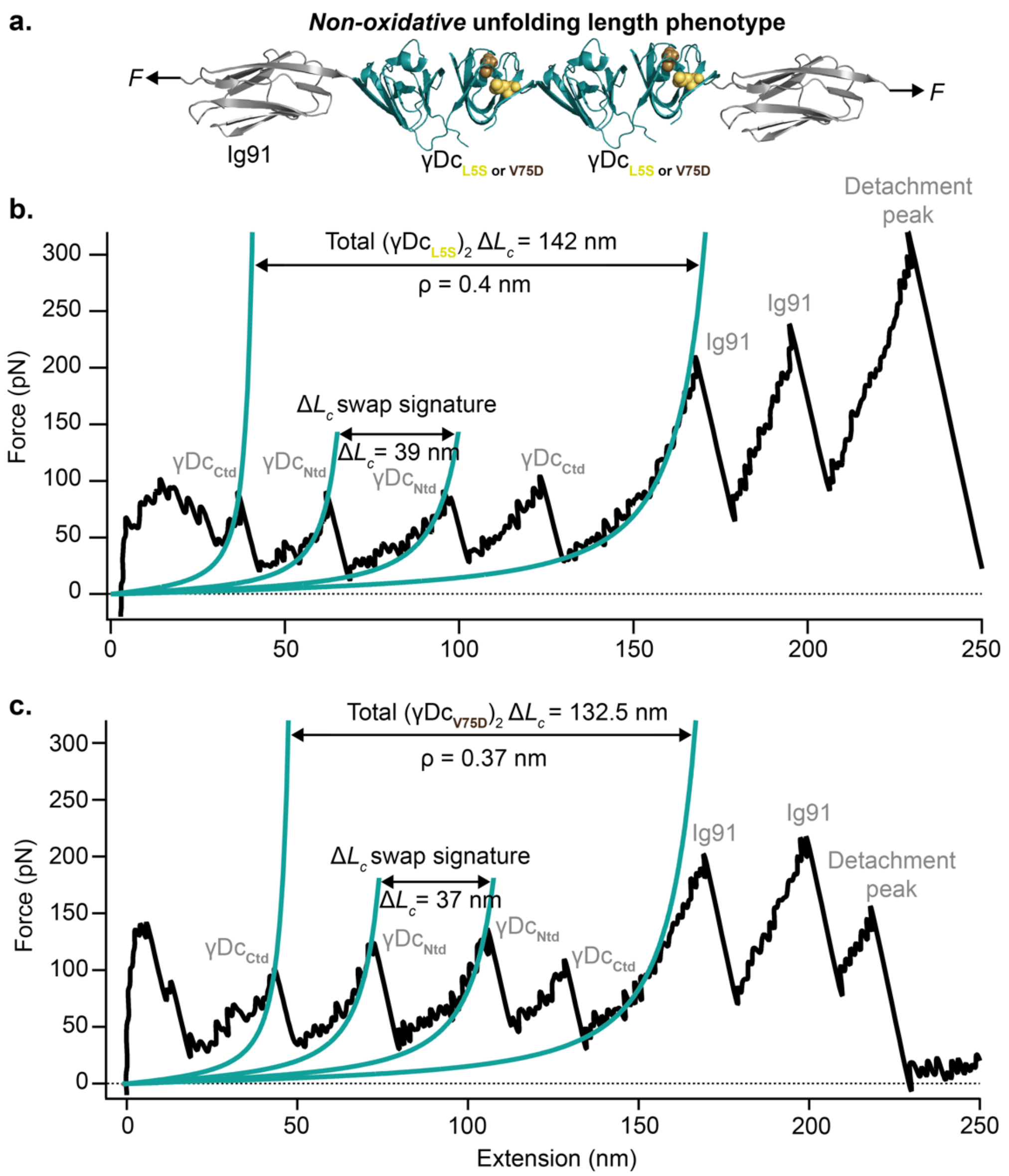

Supplementary figure 11. Domain swapped (non-oxidative) conformation in Ig91-(γDc$_{L5S\ or\ V75D}$)$_2$-Ig91 polyprotein mutants. a. Polyprotein constructs harbouring the congenital cataract point mutation (L5S and V75D respectively). Representative force vs extension unfolding trajectories **b.** Both Ig91-(γDc$_{L5S}$)$_2$-Ig91 and **c.** Ig91-(γDc$_{V75D}$)$_2$-Ig91 polyproteins display non-oxidative dimerization through Ntd exchange.

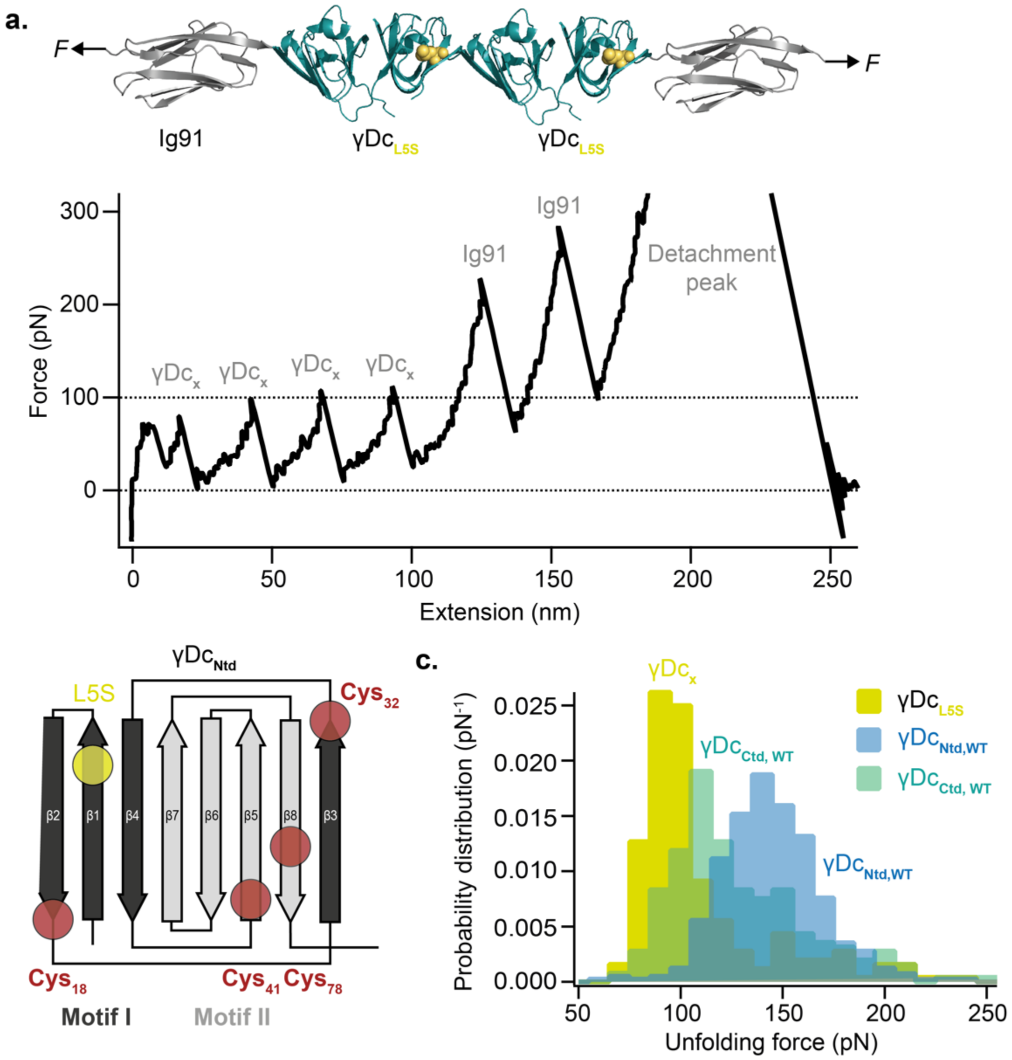

Supplementary figure 12. The L5S point-mutation reduces the mechanical stability of γDc$_{Ntd}$. a. Pulling on the Ig91-(γDc$_{L5S}$)$_2$-Ig91 polyprotein results in the initial unfolding of up to 4 similar peaks corresponding to the unfolding of the Ntds and the Ctds, with no noticeable difference in their mechanical stability (~95 pN). **b.** Location of the L5S within the Ntd structure. **c.** Comparison of the measured unfolding force corresponding to the unfolding of the γDc$_{L5S}$ (including both the Ntd and Ctd domains, as they mechanically undistinguishable) (yellow) with the unfolding forces obtained after examining each of the two terminal domains of the wild-type γDc protein individually (the green histogram corresponds to the unfolding forces of the Ctd, and the blue histogram to the Ntd).

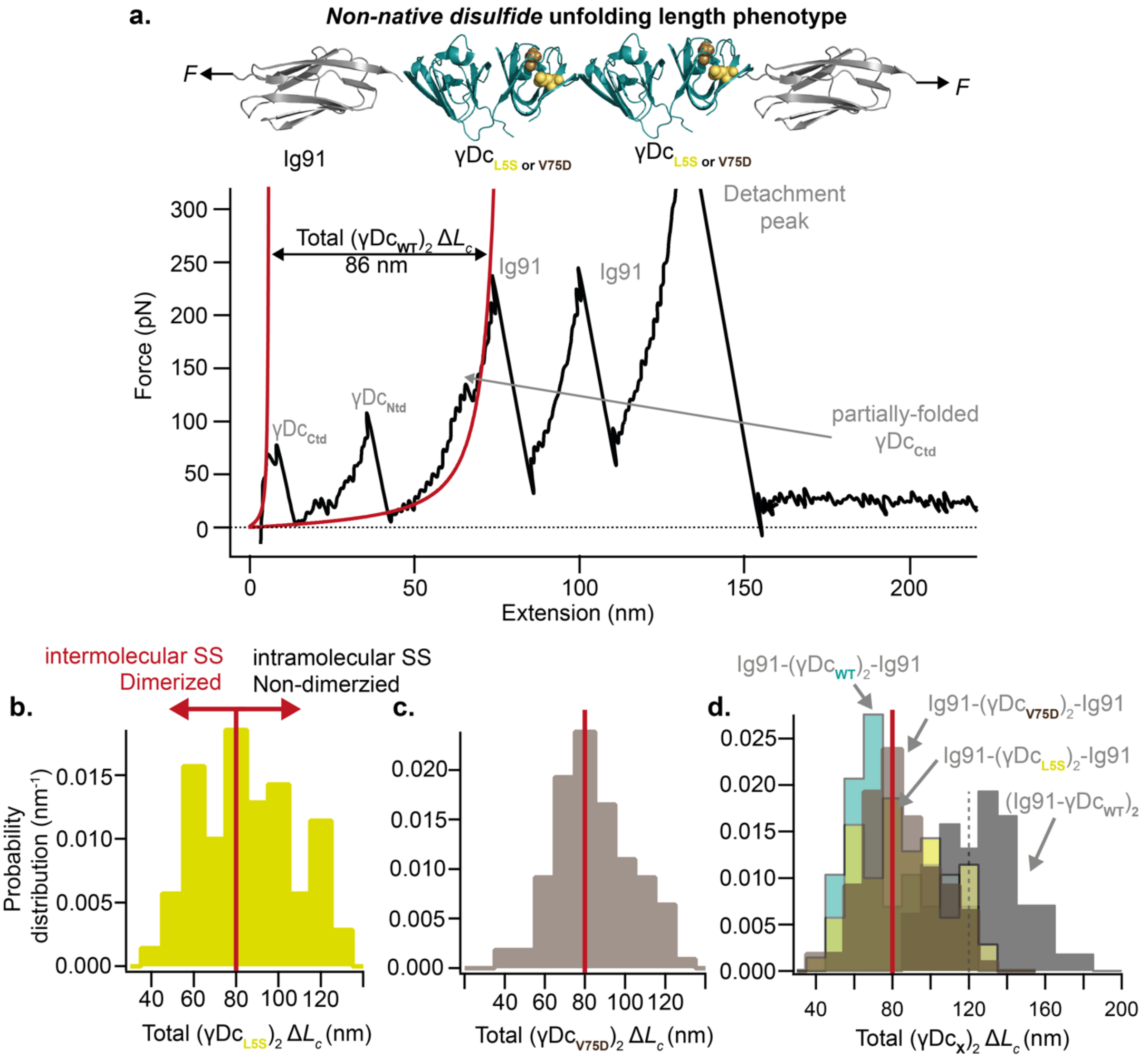

Supplementary figure 13. Mechanical unfolding of individual Ig91-(γDc$_x$)$_2$-Ig91 captures non-native intra- and intermolecular non-native disulfide bonds. a. Pulling on a Ig91-(γDc$_{WT}$)$_2$-Ig91 polyprotein results in an initial extension shorter than expected (~120 nm). This shortening fingerprints the formation of non-native disulfide bridges. **b.** Histogram showing the associated total (γDc$_{L5S}$)$_2$ ΔL_c (n=70). The red line represents the cut off (Fig. 3c) between oxidative dimerization (left) and the formation of oxidative monomeric conformations (right). **e.** Analogous histogram corresponding to the Ig91-(γDc$_{V75D}$)$_2$-Ig91 polyprotein (n=109), **f.** Histogram comparing the total (γDc$_X$)$_2$ ΔL_c measured when pulling Ig91-(γDc$_{WT,\ L5S\ and\ V75D}$)$_2$-Ig91 compared to (Ig91-γDc$_{WT}$)$_2$ (grey).

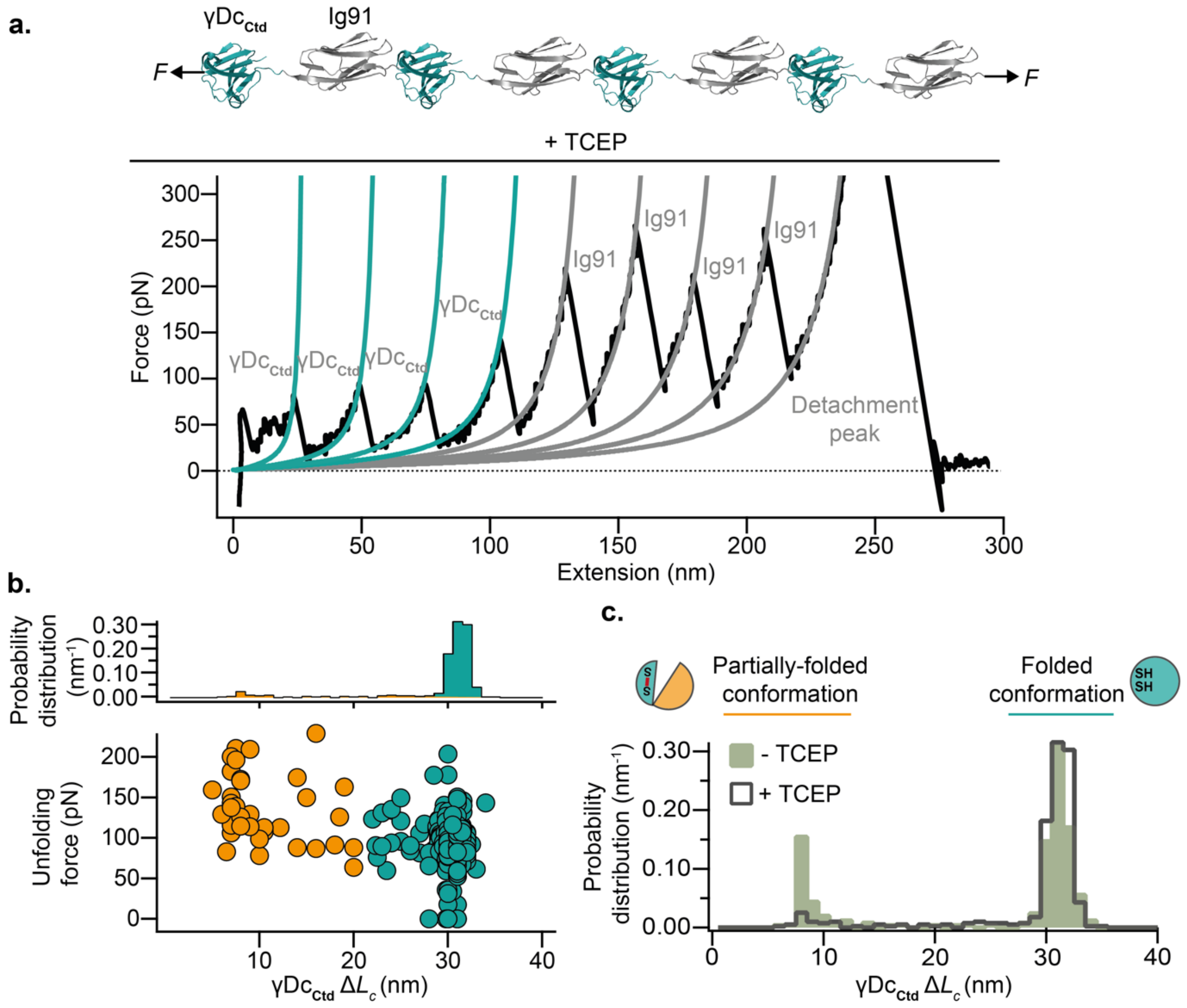

Supplementary figure 14. TCEP hinders the formation of γDC$_{Ctd}$ partially-folded conformations. a. Representative unfolding trajectory of a single (Ig91- γDc$_{Ctd}$)$_4$ polyprotein in the presence of 4 mM of TCEP, displaying the unfolding of the full Ctd length (turquoise WLC fits) followed by the unfolding of the four Ig91 (grey WLC fits). **b.** Scatterplot displaying the γDc$_{Ctd}$ increment in contour length/unfolding force relationship, accompanied by the relative frequency of each unfolding event (top) (*n*=396 unfolding events). **c.** Histogram comparing the frequency of the γDc$_{Ctd}$ increment in contour-length in the presence and absence of TCEP.

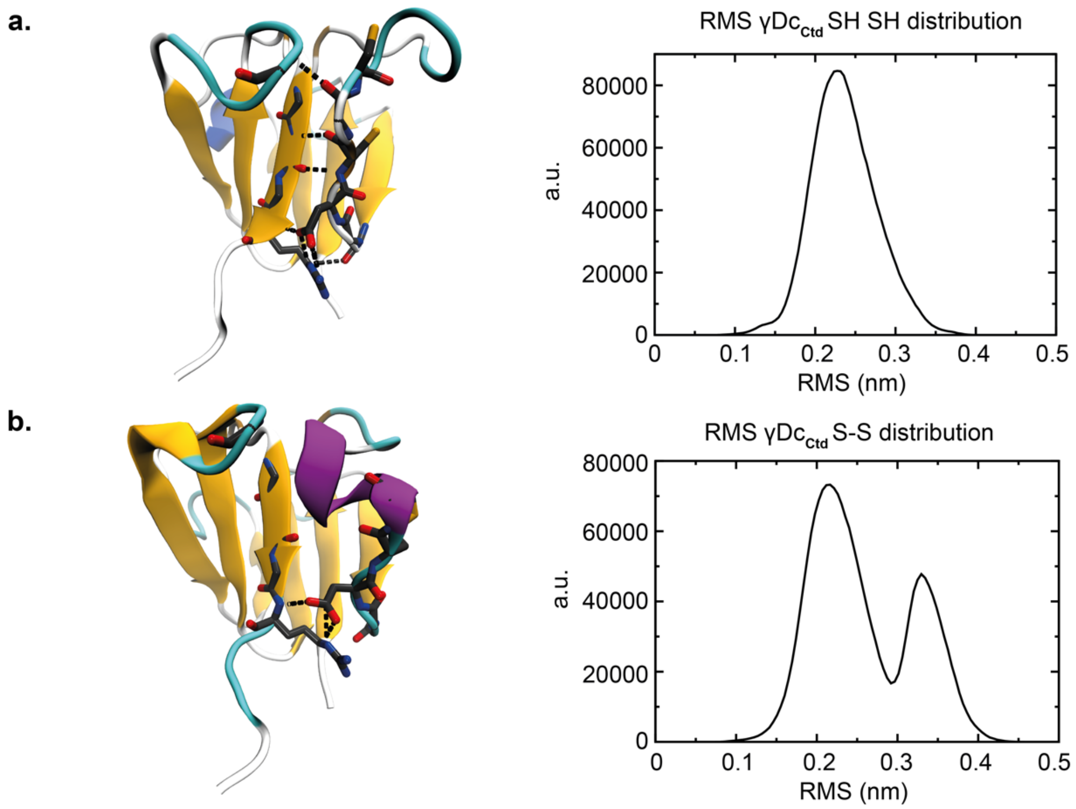

Supplementary figure 15. A non-native disulfide bond between cys$^{109\text{-}111}$ alters the γDC$_{Ctd}$ conformation. a. Pairwise RMSD distribution from the MD simulations exploring the conformational space of γDC$_{Ctd}$ at 300 K show a unique cluster close to the native structure in the absence of a disulfide bridge between cysteines 109 and 111. b. Upon non-native disulfide formation, a second cluster (~17%) where some native interactions are disrupted is also observed.

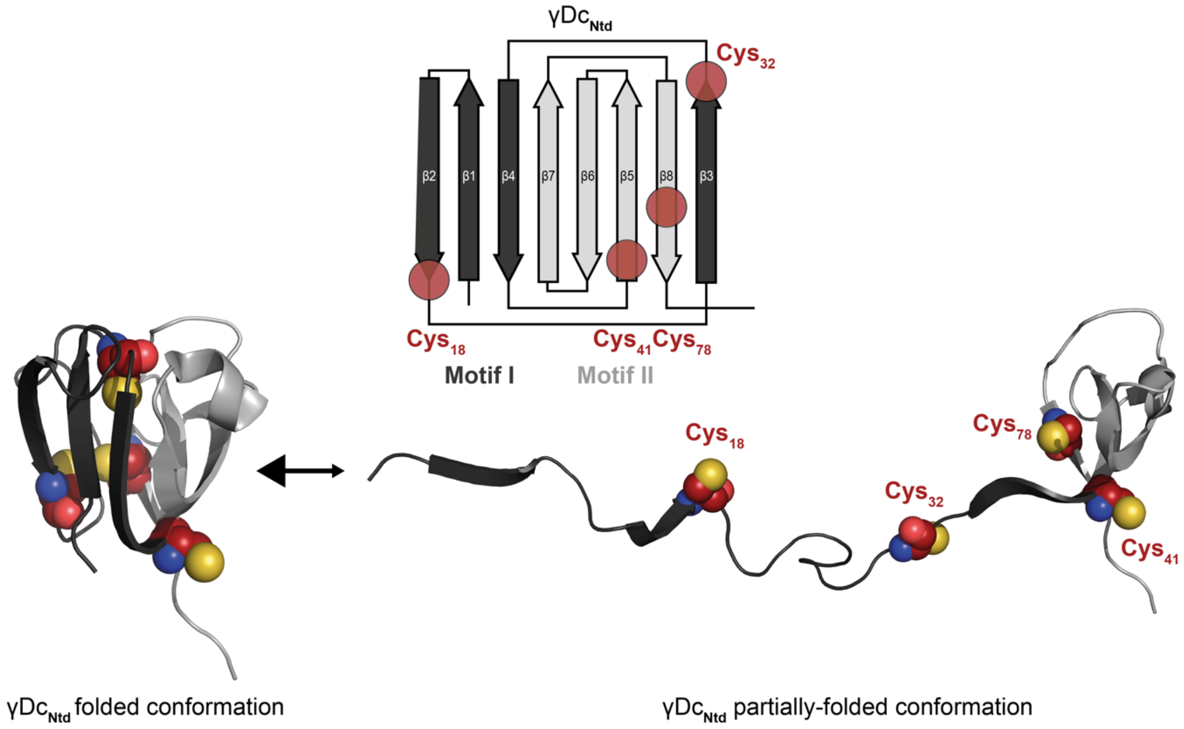

Supplementary figure 16. The putative structure of the γDc$_{Ntd}$ partially folded conformation. a. Schematic representation of the 2 Greek key motifs of γDc$_{Ntd}$ (with the four cysteines represented as red circles). **b.** Cartoon representation of the folded γDc$_{Ntd}$ (left) and the plausible Ntd partially folded conformation (right) whereby the Greek key motif II (light grey) remains folded while the first Greek key motif (black) unfolds, hence exposing cys^{18}, cys^{32} and cys^{41} to the environment.

References

(1) Schlierf, M.; Li, H.; Fernandez, J. M. The Unfolding Kinetics of Ubiquitin Captured with Single-Molecule Force-Clamp Techniques. *P Natl Acad Sci Usa* 2004, *101* (19), 7299–7304. https://doi.org/10.1073/pnas.0400033101.

(2) Popa, I.; Kosuri, P.; Alegre-Cebollada, J.; Garcia-Manyes, S.; Fernandez, J. M. Force Dependency of Biochemical Reactions Measured by Single-Molecule Force-Clamp Spectroscopy. *Nat Protoc* 2013, *8* (7), 1261–1276. https://doi.org/10.1038/nprot.2013.056.

(3) Basak, A.; Bateman, O.; Slingsby, C.; Pande, A.; Asherie, N.; Ogun, O.; Benedek, G. B.; Pande, J. High-Resolution X-Ray Crystal Structures of Human ΓD Crystallin (1.25Å) and the R58H Mutant (1.15Å) Associated with Aculeiform Cataract. *J Mol Biol* 2003, *328* (5), 1137–1147. https://doi.org/10.1016/s0022-2836(03)00375-9.

(4) Pettersen, E. F.; Goddard, T. D.; Huang, C. C.; Couch, G. S.; Greenblatt, D. M.; Meng, E. C.; Ferrin, T. E. UCSF Chimera—A Visualization System for Exploratory Research and Analysis. *J Comput Chem* 2004, *25* (13), 1605–1612. https://doi.org/10.1002/jcc.20084.

(5) Abraham, M. J.; Murtola, T.; Schulz, R.; Páll, S.; Smith, J. C.; Hess, B.; Lindahl, E. GROMACS: High Performance Molecular Simulations through Multi-Level Parallelism from Laptops to Supercomputers. *Softwarex* 2015, *1*, 19–25. https://doi.org/10.1016/j.softx.2015.06.001.

(6) Robustelli, P.; Piana, S.; Shaw, D. E. Developing a Molecular Dynamics Force Field for Both Folded and Disordered Protein States. *Proc National Acad Sci* 2018, *115* (21), 201800690. https://doi.org/10.1073/pnas.1800690115.

(7) Tribello, G. A.; Bonomi, M.; Branduardi, D.; Camilloni, C.; Bussi, G. PLUMED 2: New Feathers for an Old Bird. *Comput Phys Commun* 2014, *185* (2), 604–613. https://doi.org/10.1016/j.cpc.2013.09.018.

(8) Wang, L.; Friesner, R. A.; Berne, B. J. Replica Exchange with Solute Scaling: A More Efficient Version of Replica Exchange with Solute Tempering (REST2). *J Phys Chem B* 2011, *115* (30), 9431–9438. https://doi.org/10.1021/jp204407d.

(9) Humphrey, W.; Dalke, A.; Schulten, K. VMD: Visual Molecular Dynamics. *J Mol Graphics* 1996, *14* (1), 33–38. https://doi.org/10.1016/0263-7855(96)00018-5.
